# Global, regional, and national differences in the incidence and mortality of digestive congenital anomalies from 1990 to 2021, with projections for future trends

**DOI:** 10.3389/fpubh.2025.1640700

**Published:** 2025-10-14

**Authors:** Baihui Yan, Wei Li, Peng Li

**Affiliations:** ^1^Department of Anesthesiology, The Second Affiliated Hospital of Xi’an Jiaotong University, Xi’an, China; ^2^Department of Pediatric Surgery, The Second Affiliated Hospital of Xi’an Jiaotong University, Xi’an, China

**Keywords:** digestive congenital anomalies, Global Burden of Disease, incidence, mortality, ARIMA projection

## Abstract

**Introduction:**

Digestive congenital anomalies (DCA) remain a major yet unevenly distributed cause of death and disability worldwide. We aimed to quantify global, regional, and national trends in DCA prevalence, mortality, and disability-adjusted life years (DALYs) from 1990 to 2021.

**Methods:**

We analyzed Global Burden of Disease 2021 data. Age-standardized rates and estimated annual percentage changes were calculated for five age groups: <1 year, 2–4 years, 5–14 years, 15–19 years, and 20–54 years. Future trajectories to 2036 were projected using an autoregressive integrated moving average (ARIMA) model.

**Results:**

In 2021, infants accounted for the largest share of the burden, although their absolute incidence declined by 9% relative to 1990. High–socio-demographic index (SDI) regions achieved pronounced reductions in both mortality and DALYs, whereas low-SDI regions showed rising trends. Projections indicate a continued global increase in prevalence across all age groups, alongside further declines in infant and child mortality.

**Discussion:**

Persistent inequities underscore the need for strengthened maternal–neonatal services, early surgical access, and targeted resource allocation in low-income settings. This integrated epidemiological overview provides an evidence base for prioritizing DCA within national child-health agendas and for monitoring progress toward Sustainable Development Goal targets.

## 1 Introduction

Congenital digestive tract anomalies (CDTA) are prevalent congenital disorders in neonates with substantial clinical significance, typically diagnosed at or shortly after birth (1–3). CDTA primarily comprise five major categories: esophageal anomalies (e.g., esophageal atresia and tracheoesophageal fistula), gastric and duodenal anomalies (e.g., pyloric hypertrophic stenosis and duodenal atresia), small bowel malformations (e.g., jejunal and ileal atresia and intestinal malrotation), large bowel malformations (e.g., Hirschsprung’s disease and colonic atresia), and anorectal malformations (e.g., anal atresia and rectal fistula) (4–7). The global incidence of CDTA is estimated to range between 5.0 and 25.0 cases per 10,000 live births. These anomalies can result in severe clinical symptoms in neonates, including vomiting, abdominal distension, feeding difficulties, and abnormal bowel movements, often necessitating urgent surgical intervention (8).

For instance, congenital esophageal atresia (EA), frequently accompanied by tracheoesophageal fistula (TEF), represents one of the most prevalent forms of CDTA (9). EA obstructs the normal passage of food from the mouth to the stomach, severely impairing the neonate’s ability to feed and absorb nutrients (10). Without timely surgical intervention, postnatal mortality in infants with EA can reach up to 90%. Although surgery markedly improves survival, postoperative complications, such as gastroesophageal reflux (41.4%), dysphagia (27.6%), and esophagitis (12.4%) continue to significantly affect long-term health outcomes (11). These high complication rates underscore the need for long-term medical surveillance and postoperative care in EA/TEF patients, highlighting the necessity for further optimization of treatment strategies (12).

In recent decades, advancements in imaging technology, minimally invasive surgery, and perioperative care have led to substantial reductions in mortality associated with congenital digestive tract anomalies (CDTA) (13). For instance, the Global Burden of Disease (GBD) study reported that between 2010 and 2019, CDTA-related mortality in many high-income countries (HIC) decreased significantly, with an average annual reduction of 2.7%. However, this trend was less pronounced in low- and middle-income countries (LMIC), contributing to disparities in disease burden across regions (8). Despite these findings, comprehensive analyses of GBD 2021 data on CDTA are currently lacking in major databases such as PubMed and Google Scholar. This underscores the need for further research to fully elucidate the global burden of CDTA and its regional variations.

Despite significant global advances in the diagnosis and treatment of CDTA, these improvements have been largely confined to high-income countries (HIC) (14). These nations typically benefit from advanced medical infrastructure, highly trained surgical teams, and comprehensive prenatal screening programs, resulting in significantly better survival rates and long-term outcomes for affected children compared to low- and middle-income countries (LMIC). This substantial disparity is primarily attributed to a severe shortage of medical resources, a lack of specialized medical personnel, and inadequate prenatal diagnostic and screening technologies in LMIC (15).

This disparity underscores the substantial global inequity in medical resource distribution, emphasizing the urgent need to improve medical infrastructure and strengthen professional capacity in low- and middle-income countries (LMICs) (16). Therefore, conducting in-depth analyses of prevalence and mortality rates across regions using Global Burden of Disease (GBD) data is essential for developing more targeted public health strategies. Such analyses will help identify high-risk factors, inform resource allocation, and promote global health equity.

## 2 Method

### 2.1 Data source

The data for this study were obtained from the Global Burden of Disease (GBD) 2021 database, accessed via the GBD Results Tool (17). Primary inputs to GBD include vital registration (VR), verbal autopsy, health-facility records, surveys, and the published literature, which vary in coverage and quality across settings, particularly in low-SDI regions where early neonatal deaths may be under-registered or misclassified (18). GBD produces model-based estimates with 95% uncertainty intervals (UIs); therefore, we interpret results with UIs rather than point estimates alone.

### 2.2 Data collection

The GBD 2021 study reports incidence, mortality, and disability-adjusted life years (DALYs) for 369 diseases and injuries across 204 countries and territories (1990–2021). For digestive congenital anomalies (DCA), we extracted prevalence, mortality, and DALYs from 1990 to 2021 at global, regional, and national levels, stratified by age groups <1 year, 2–4 years, 5–14 years, 15–19 years, and 20–54 years and by SDI quintiles. We define DCA to include esophageal atresia, intestinal atresia/stenosis, anorectal malformations, Hirschsprung disease, gastroschisis, omphalocele, and biliary atresia, mapped to GBD cause IDs. Our estimates reflect live-birth incidence/prevalence; prenatal detection and, where lawful, termination of pregnancy for fetal anomaly (TOPFA) are not captured in live-birth records and may lower recorded levels in high-SDI settings. Potential time-varying changes in the birth prevalence of DCA and the modifying effects of co-morbidities (e.g., prematurity, sepsis, congenital heart disease, malnutrition) are considered (19).

### 2.3 Statistical analysis

Prevalence, mortality, DALYs, and their corresponding rates are the key metrics used to assess the burden of digestive congenital anomalies (DCA) (20). This study applied the GBD algorithms to calculate these rates per 100,000 population, reporting 95% uncertainty intervals. To analyze time trends in DCA, the mean estimated annual percentage change (EAPC) was calculated using a linear regression model. A negative value for both the EAPC and the upper limit of its 95% confidence interval indicates a declining trend, while a positive value for both the EAPC and the lower limit of its confidence interval indicates an increasing trend. All statistical analyses were performed using RStudio (version 4.2.0) and R software (version 4.4.1) (21).

### 2.4 Future trend prediction

To further observe the prevalence, mortality, and DALYs among the five aforementioned populations, the future trends for the next 15 years were predicted using the autoregressive integrated moving average (ARIMA) model (22, 23). ARIMA consists of the autoregressive (AR) and moving average (MA) components. It assumes that the data series is a transient random variable, where autocorrelation can be characterized by the ARIMA model, enabling predictions of future values based on past values. The formula is expressed as follows:


$$y_{t}=\delta +\phi_{1}y_{t-1}+\ldots +\phi_{p}y_{t-p}+\theta_{1}\varepsilon_{t-1}+\ldots +\theta_{q}\varepsilon_{t-q}+\varepsilon_{t}$$

Where $y_{t}$ represents the time series at different time points, and the right-hand side includes the lagged values of $y_{t}$ and the lagged errors.

For each outcome–age stratum, we fitted annual univariate ARIMA models using AICc/BIC–guided auto.arima, determined the minimum differencing via ADF/KPSS, and accepted models only after residual diagnostics (Ljung–Box, ACF/PACF). We benchmarked against ETS and naïve forecasts using rolling-origin cross-validation (horizons 1, 3, 5).

## 3 Results

### 3.1 Global trends (global)

#### 3.1.1 Prevalence

Global trend data from 2021 reveal significant variations in the number of digestive congenital anomalies (DCA) cases across age groups. Approximately 368,774 DCA cases were reported in infants under 1 year of age (95% uncertainty interval [UI]: 288,416–472,689), 624,244 cases in toddlers aged 2–4 years (95% UI: 484,083–786,655), approximately 923,306 cases in children aged 5–14 years (95% UI: 706,675–1,183,390), 212,564 cases (95% UI: 160,079–269,112) in adolescents aged 15–19 years, and approximately 641,013 cases (95% UI: 503,503–815,586) in adults aged 20–54 years.

Between 1990 and 2021, the number of DCA cases in infants aged 0–4 years exhibited a declining trend, with a reduction of 9% (95% UI: −17 to 1%). The corresponding prevalence rates also decreased, from 317.35 (95% UI: 240.03–404.66) and 175.66 (95% UI: 133.98–220.01) to 291.07 (95% UI: 227.65–373.09) and 154.87 (95% UI: 120.1–195.17), respectively. The estimated annual percentage changes (EAPC) were −0.08 (95% UI: −0.17 to 0.02) and −0.3 (95% UI: −0.36 to −0.23).

In contrast, the number of DCA cases in children aged 5–14 years, adolescents aged 15–19 years, and adults aged 20–54 years exhibited an upward trend over the same period, with increases of 5, 13, and 52%, respectively. The number of cases rose from 877,609 (95% UI: 660,411–1,104,840), 187,544 (95% UI: 139,084–243,463), and 422,030 (95% UI: 328,040–543,626) to 923,306 (95% UI: 706,675–1,183,390), 212,564 (95% UI: 160,079–269,112), and 641,013 (95% UI: 503,503–815,586), respectively. Despite the increase in the number of cases, the overall prevalence in these age groups declined, from 78.41 (95% UI: 59.01–98.72), 36.11 (95% UI: 26.78–46.87), and 17.56 (95% UI: 13.65–22.62) to 68.21 (95% UI: 52.2–87.42), 34.07 (95% UI: 25.65–43.13), and 17.01 (95% UI: 13.36–21.64), respectively. The estimated annual percentage changes (EAPC) were −0.43 (95% UI: −0.5 to −0.37), −0.33 (95% UI: −0.42 to −0.23), and −0.35 (95% UI: −0.47 to −0.23), respectively.

**Figure 1:**
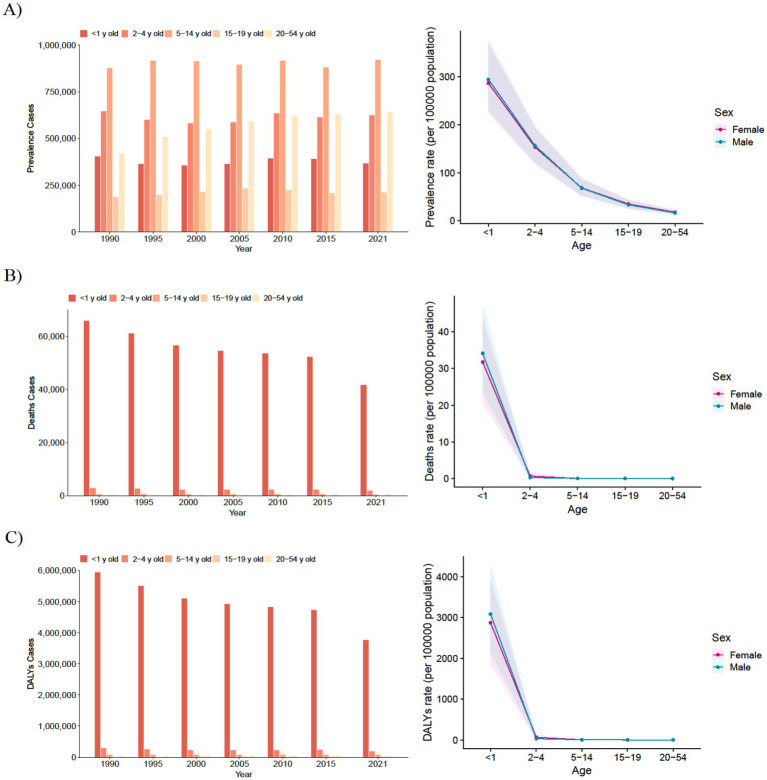
Trends in prevalence of congenital malformations of the gastrointestinal tract, deaths and DALYs in five different age groups, 1990–2021. **(A)** Trends in prevalence cases and prevalence rate. **(B)** Trends in deaths cases and deaths rate. **(C)** Trends in DALYs cases and DALYs rate.

Between 1990 and 2019, the most substantial increase in prevalence occurred in adults aged 20–54 years, whereas the highest disease burden was observed in children aged 5–14 years. No significant differences in prevalence changes were found between males and females (Figure 1A; Table 1 and Supplementary Table S1).

**Table 1:** Global and regional mortality data for all ages, children younger than 1 year, and age-standardized mortality rates (1990–2021).

	Number of deaths (all ages)			Mortality per 100,000 children younger than 1 year			Age-standardized mortality per 100,000 individuals		
	1990	2021	Percentage change, 1990–2021	1990	2021	Percentage change, 1990–2021	1990	2021	Percentage change, 1990–2021
Global	75,201 (41,786–116,847)	48,483 (36,340–60,375)	−0.36 (−0.58 to 0.16)	51.71 (29.97–78.94)	33.03 (24.83–41.75)	−0.36 (−0.58 to 0.13)	1.19 (0.66–1.85)	0.77 (0.58–0.96)	−0.35 (−0.57 to 0.17)
SDI									
Low SDI	16,554 (5,516–28,649)	19,343 (12,834–26,603)	0.17 (−0.26 to 1.57)	68.11 (25.06–115.77)	47.28 (32.49–66.04)	−0.31 (−0.56 to 0.44)	1.66 (0.55–2.89)	1.15 (0.77–1.58)	−0.31 (−0.56 to 0.52)
Low-middle SDI	21,883 (11,013–37,274)	15,795 (11,175–20,713)	−0.28 (−0.58 to 0.54)	53.75 (27.88–90.44)	37.13 (26.43–49.6)	−0.31 (−0.59 to 0.41)	1.21 (0.61–2.07)	0.85 (0.6–1.11)	−0.3 (−0.59 to 0.48)
Middle SDI	22,233 (13,532–36,170)	9,608 (7,268–12,364)	−0.57 (−0.74 to −0.15)	48.95 (30.54–78.16)	26.16 (19.67–34.3)	−0.47 (−0.68 to 0.04)	1.12 (0.68–1.82)	0.6 (0.45–0.78)	−0.46 (−0.68 to 0.06)
High-middle SDI	11,464 (7,201–16,772)	2,557 (1,862–3,285)	−0.78 (−0.87 to −0.6)	56.67 (35.75–81.64)	18.16 (13.2–23.48)	−0.68 (−0.81 to −0.42)	1.29 (0.81–1.89)	0.42 (0.31–0.55)	−0.67 (−0.81 to −0.4)
High SDI	3,004 (2,596–3,593)	1,144 (907–1,357)	−0.62 (−0.72 to −0.54)	20.26 (17.38–24.45)	8.48 (6.49–10.17)	−0.58 (−0.7 to −0.49)	0.49 (0.42–0.58)	0.21 (0.16–0.25)	−0.57 (−0.7 to −0.48)
Region									
Andean Latin America	1,070 (556–1,727)	606 (425–805)	−0.43 (−0.69 to 0.17)	86.22 (45.84–138.7)	44.73 (31.49–59.86)	−0.48 (−0.72 to 0.05)	1.94 (1.01–3.14)	1.01 (0.71–1.34)	−0.48 (−0.72 to 0.07)
Australasia	81 (71–99)	27 (21–42)	−0.66 (−0.74 to −0.52)	21.47 (18.93–26.07)	6.14 (4.68–9.38)	−0.71 (−0.79 to −0.59)	0.52 (0.46–0.64)	0.15 (0.11–0.23)	−0.71 (−0.78 to −0.59)
Caribbean	791 (455–1,300)	488 (288–780)	−0.38 (−0.59 to 0)	78.78 (47.05–122.85)	55.21 (33.41–89.08)	−0.3 (−0.53 to 0.15)	1.86 (1.07–3.05)	1.27 (0.75–2.03)	−0.32 (−0.54 to 0.11)
Central Asia	740 (575–932)	614 (485–789)	−0.17 (−0.37 to 0.12)	35.81 (28.03–45.19)	28.03 (22.15–35.63)	−0.22 (−0.4 to 0.06)	0.79 (0.62–0.99)	0.62 (0.49–0.8)	−0.21 (−0.4 to 0.06)
Central Europe	909 (682–1,175)	147 (115–186)	−0.84 (−0.89 to −0.76)	47.83 (35.6–62.26)	12.23 (9.54–15.27)	−0.74 (−0.83 to −0.62)	1.08 (0.81–1.39)	0.28 (0.22–0.35)	−0.74 (−0.83 to −0.62)
Central Latin America	2,648 (2,281–3,288)	1,982 (1,478–2,561)	−0.25 (−0.5 to 0.04)	49.33 (42.61–61.29)	44.24 (32.77–57.58)	−0.1 (−0.41 to 0.26)	1.13 (0.97–1.4)	1.03 (0.77–1.33)	−0.09 (−0.39 to 0.27)
Central Sub-Saharan Africa	1,835 (450–3,571)	1,641 (883–2,859)	−0.11 (−0.47 to 1.24)	67.13 (17.58–129.15)	33.58 (18.22–60.16)	−0.5 (−0.7 to 0.17)	1.58 (0.4–3.06)	0.78 (0.43–1.36)	−0.5 (−0.72 to 0.26)
East Asia	14,416 (7,678–26,231)	2,595 (1,639–3,815)	−0.82 (−0.92 to −0.58)	54.42 (29.71–96.56)	18.6 (11.61–27.57)	−0.66 (−0.84 to −0.22)	1.26 (0.67–2.29)	0.43 (0.27–0.64)	−0.66 (−0.84 to −0.2)
Eastern Europe	1,792 (1,429–2,101)	323 (246–429)	−0.82 (−0.88 to −0.73)	53.6 (42.45–63.06)	14.4 (10.68–19.12)	−0.73 (−0.82 to −0.6)	1.21 (0.96–1.42)	0.34 (0.25–0.45)	−0.72 (−0.81 to −0.59)
Eastern Sub-Saharan Africa	6,796 (1,586–13,993)	6,584 (3,660–10,676)	−0.03 (−0.44 to 1.59)	70.79 (17.75–144.44)	43.1 (23.62–70.99)	−0.39 (−0.64 to 0.51)	1.69 (0.41–3.48)	1.02 (0.59–1.66)	−0.39 (−0.65 to 0.6)
High-income Asia Pacific	468 (364–598)	106 (80–138)	−0.77 (−0.85 to −0.68)	17.57 (13.65–22.3)	5.39 (3.87–7.62)	−0.69 (−0.8 to −0.54)	0.46 (0.36–0.59)	0.15 (0.11–0.2)	−0.68 (−0.8 to −0.53)
High-income North America	752 (693–948)	481 (376–554)	−0.36 (−0.55 to −0.25)	13.92 (12.78–17.84)	9.19 (7.07–10.86)	−0.34 (−0.55 to −0.21)	0.34 (0.31–0.42)	0.22 (0.17–0.26)	−0.33 (−0.53 to −0.21)
North Africa and Middle East	10,295 (3,617–20,239)	4,279 (2,927–5,630)	−0.58 (−0.76 to 0)	88.85 (31.82–169.81)	32.1 (22.22–42.34)	−0.64 (−0.79 to −0.14)	1.99 (0.7–3.92)	0.74 (0.5–0.97)	−0.63 (−0.79 to −0.11)
Oceania	32 (12–73)	59 (22–144)	0.83 (0.08 to 1.98)	14.43 (5.04–32.34)	13.67 (5.06–33.47)	−0.05 (−0.45 to 0.57)	0.31 (0.11–0.69)	0.29 (0.11–0.71)	−0.05 (−0.44 to 0.55)
South Asia	15,734 (6,820–28,790)	9,836 (5,622–16,429)	−0.37 (−0.65 to 0.42)	45.13 (19.83–82.32)	29.59 (16.52–49.72)	−0.34 (−0.64 to 0.44)	0.98 (0.43–1.79)	0.64 (0.37–1.08)	−0.34 (−0.63 to 0.49)
Southeast Asia	5,255 (2,440–9,468)	3,383 (2,262–4,627)	−0.36 (−0.62 to 0.5)	37.62 (18.58–65.94)	26.75 (17.42–37.59)	−0.29 (−0.58 to 0.59)	0.9 (0.42–1.62)	0.62 (0.41–0.85)	−0.31 (−0.59 to 0.61)
Southern Latin America	491 (403–625)	246 (188–306)	−0.5 (−0.64 to −0.34)	44.24 (35.74–56.38)	28.48 (21.32–36.08)	−0.36 (−0.54 to −0.14)	0.97 (0.79–1.23)	0.64 (0.48–0.8)	−0.34 (−0.52 to −0.12)
Southern Sub-Saharan Africa	480 (331–609)	410 (229–650)	−0.14 (−0.45 to 0.21)	26 (17.72–34.6)	21.52 (11.64–35.58)	−0.17 (−0.45 to 0.13)	0.64 (0.44–0.81)	0.52 (0.29–0.83)	−0.18 (−0.48 to 0.16)
Tropical Latin America	1,649 (1,349–2,091)	1,370 (1,091–1,717)	−0.17 (−0.41 to 0.14)	45.9 (37.45–58.21)	35.27 (27.79–44.62)	−0.23 (−0.46 to 0.07)	1.03 (0.84–1.3)	0.81 (0.64–1.01)	−0.21 (−0.44 to 0.09)
Western Europe	1,166 (979–1,503)	404 (339–498)	−0.65 (−0.75 to −0.56)	20.93 (17.55–27.13)	7.49 (6.15–9.19)	−0.64 (−0.74 to −0.54)	0.5 (0.42–0.65)	0.18 (0.15–0.22)	−0.63 (−0.74 to −0.54)
Western Sub-Saharan Africa	7,802 (2,485–12,327)	12,902 (8,053–17,710)	0.65 (0.17 to 2.69)	72.91 (27.11–103.3)	61.31 (40.31–85.85)	−0.16 (−0.38 to 0.69)	1.98 (0.65–3.13)	1.58 (1.01–2.17)	−0.2 (−0.45 to 0.77)

#### 3.1.2 Mortality rate

Over the 32-year period from 1990 to 2021, global deaths related to digestive congenital anomalies (DCA) in infants under 1 year, toddlers aged 2–4 years, and children aged 5–14 years decreased by 37, 36, and 12%, respectively. Specifically, infant deaths declined from 66,062 (95% uncertainty interval [UI]: 38,287–100,849) to 41,842 (95% UI: 31,457–52,893); toddler deaths from 3,037 (95% UI: 629–6,799) to 1,951 (95% UI: 751–3,629); and deaths in children aged 5–14 years from 672 (95% UI: 425–969) to 594 (95% UI: 421–836). In contrast, deaths among adolescents aged 15–19 years and adults aged 20–54 years increased by 10 and 58%, respectively. Adolescent deaths rose from 130 (95% UI: 92–178) to 144 (95% UI: 94–210), and adult deaths increased from 431 (95% UI: 325–532) to 680 (95% UI: 487–956).

In terms of mortality rates, an overall downward trend was observed between 1990 and 2021 across all age groups, except for adults aged 20–54 years. Infant mortality rates declined from 51.71 (95% UI: 29.97–78.94) to 33.03 (95% UI: 24.83–41.75), young child mortality from 0.83 (95% UI: 0.17–1.85) to 0.48 (95% UI: 0.19–0.9), child mortality from 0.06 (95% UI: 0.04–0.09) to 0.04 (95% UI: 0.03–0.06), and adolescent mortality from 0.03 (95% UI: 0.02–0.03) to 0.02 (95% UI: 0.02–0.03). The corresponding mean estimated annual percentage changes (EAPC) were −1.32 (95% UI: −1.37 to −1.27), −1.35 (95% UI: −1.48 to −1.21), −0.65 (95% UI: −0.77 to −0.53), and −0.23 (95% UI: −0.27 to −0.20) (see Supplementary Table S2).

Although deaths among individuals aged 15–19 years and 20–54 years increased significantly, the number of deaths in infants under 1 year far exceeded that of other age groups, being approximately 100 times higher. Similar to the prevalence rate, no significant difference was observed in the change in the number of deaths between males and females across the five age groups (see Figure 1B).

#### 3.1.3 DALYs

From 1990 to 2021, there was a significant decline in disability-adjusted life years (DALYs) related to congenital digestive tract anomalies (DCA) among infants under 1 years, young children aged 2–4 years, and children aged 5–14 years globally. DALYs for infants declined from 5,954,515.4 (95% uncertainty interval [UI]: 3,459,155.44–9,080,801.09) in 1990 to 3,777,891.23 (95% UI: 2,846,143.52–4,774,021.88), representing a 37% decrease. DALYs for young children aged 2–4 years decreased from 293,581.9 (95% UI: 84,002.22–619,337.82) to 198,023.11 (95% UI: 91,135.74–338,215.2), a 33% decrease. DALYs for children aged 5–14 years fell from 93,847.97 (95% UI: 66,053.11–125,988.29) to 89,325.01 (95% UI: 67,045.69–119,318.29), a 5% decrease. The corresponding DALY rates also followed a downward trend, declining from 4661.16 (95% UI: 2707.8–7108.39), 79.87 (95% UI: 22.85–168.49), and 8.39 (95% UI: 5.9–11.26) to 2981.89 (95% UI: 2246.46–3768.14), 49.13 (95% UI: 22.61–83.91), and 6.6 (95% UI: 4.95–8.81), respectively. The corresponding mean estimated annual percentage changes (EAPC) were −1.31 (95% UI: −1.36 to −1.27), −1.23 (95% UI: −1.34 to −1.11), and −0.56 (95% UI: −0.64 to −0.48).

In contrast, the number of DALYs for adolescents aged 15–19 years and adults aged 20–54 years increased by 12 and 53%, respectively. However, the DALY rates for these two age groups decreased from 3.4 (95% UI: 2.52–4.52) and 1.75 (95% UI: 1.37–2.22) to 3.18 (95% UI: 2.33–4.36) and 1.71 (95% UI: 1.29–2.26), respectively, in 2021. The corresponding EAPCs were −0.27 (95% UI: −0.32 to −0.22) and −0.17 (95% UI: −0.23 to −0.11) (see Supplementary Table S3 and Figure 1C).

### 3.2 Regional trends in SDI

#### 3.2.1 Prevalence

**Figure 2:**
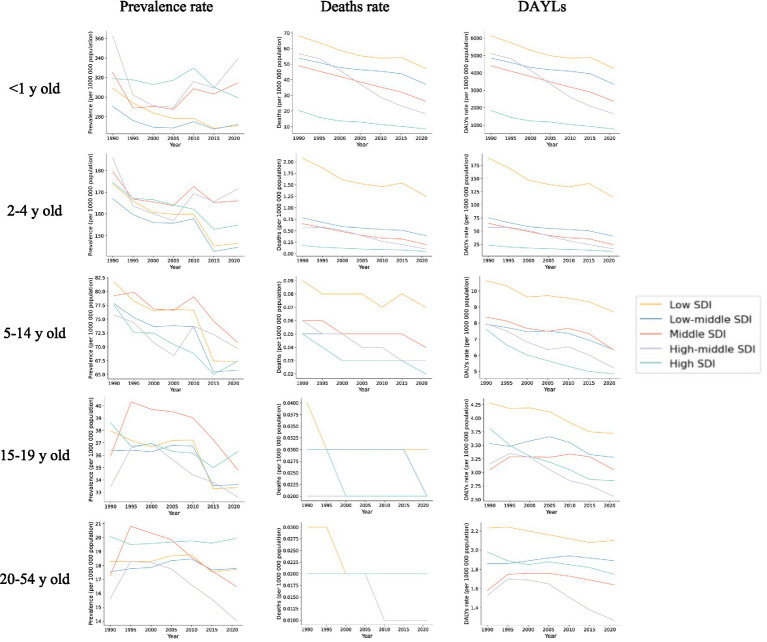
Trends in prevalence, deaths and DALYs in five socio-demographic indices (SDIs) for five different age groups, 1990–2021.

Data from 2021 show that the number of Digestive Congenital Anomalies (DCA) cases across all age groups was highest in the Middle Socio-demographic Index (SDI) region. Notably, a significant increase in cases was observed in the Low SDI region among infants under 1 year and children aged 2–4 years, with increases of 48 and 59%, respectively. Additionally, among children aged 5–14 years, adolescents aged 15–19 years, and adults aged 20–54 years, two or more SDI regions showed an upward trend in the number of prevalent cases, with the largest increases occurring in Low SDI regions, at 76, 116, and 137%, respectively (see Supplementary Table S4 and Figure 2).

#### 3.2.2 Mortality

Similar to prevalence, the number of deaths related to Digestive Congenital Anomalies (DCA) across all age groups was highest in the Middle Socio-demographic Index (SDI) region. However, the proportion of deaths among infants under 1 year was significantly higher than in the other four age groups, with the greatest increase observed in the Low SDI region, at 17%. Specifically, the number of deaths among infants under 1 year increased from 13,932.93 (95% UI: 5,125.63–23,682.03) in 1990 to 16,331.15 (95% UI: 11,222.59–22,813.05) in 2021. Nonetheless, all regions exhibited a decreasing trend in overall mortality, and the corresponding average estimated annual percentage change (EAPC) was negative across all regions. The smallest decrease occurred in the Low SDI region (−0.95, 95% UI: −1.05 to −0.86), while the largest decrease was in the High SDI region (−2.39, 95% UI: −2.52 to −2.25). Other age-specific mortality rates and their corresponding EAPCs similarly exhibited a negative trend (see Supplementary Table S5 and Figure 2).

#### 3.2.3 DALYs

From 1990 to 2021, in contrast to the global trend, DCA-related disability-adjusted life years (DALYs) across all age groups saw the largest increases in the Low Socio-demographic Index (SDI) region, rising by 17, 15, 75, 113, and 130%, respectively. Specifically, DALYs increased from 1,254,515 (95% UI: 463,849–2,129,621) among infants under 1 year, 97,269 (95% UI: 8,402–274,673) among toddlers aged 2–4 years, 14,691 (95% UI: 8,402–27,467) among children aged 5–14 years, 2,166 (95% UI: 1,475–3,043) among adolescents aged 15–19 years, and 41,144 (95% UI: 28,681–60,036) among adults aged 20–54 years in 1990. By 2021, DALYs had risen to 1,471,590 (95% UI: 1,011,966–2,052,809) for infants, 112,004 (95% UI: 30,506–227,821) for toddlers, 25,675 (95% UI: 17,852–37,186) for children, 4,611 (95% UI: 2,848–7,301) for adolescents, and 9,453 (95% UI: 5,585–15,808) for adults.

Although both the number and rate of DALYs were significantly higher in infants under 1 year and children aged 2–4 years compared to other age groups, an overall decreasing trend in DALY rates was observed across all age groups, with corresponding EAPCs being negative. For the most part, the smallest decreases were observed in Low or Low-Middle SDI regions, except for the 15–19 and 20–54 years age groups, where the smallest decreases occurred in Middle SDI regions (see Supplementary Table S6 and Figure 2).

### 3.3 Regional trends (region)

#### 3.3.1 Prevalence

In 2021, the highest prevalence of Digestive Congenital Anomalies (DCA) across all age groups in the 21 global regions was observed in South Asia. The number of cases was 268,039 (95% UI: 209,709–268,039), 53,930 (95% UI: 39,056–69,983), and 147,731 (95% UI: 113,327–189,472). Excluding the adult group aged 20–54 years, the regions with the lowest number of DCA cases across all age groups were in Australasia, with 689 (95% UI: 534–864), 1,133 (95% UI: 880–1,411), 1,617 (95% UI: 1,202–2,045), and 353 (95% UI: 262–463). The region with the lowest number of cases in adults aged 20–54 years was Oceania, with 1,142 cases (95% UI: 905–1,463).

From 1990 to 2021, the largest increase in the prevalence of DCA among infants under 1 year was observed in East Asia, with an EAPC of 1.17 (95% UI: 0.59–1.76), while the largest decrease occurred in High-income Asia Pacific, with an EAPC of −1.25 (95% UI: −1.34 to −1.16). The largest increases in prevalence across the remaining four age groups were observed in Southern Latin America, with EAPCs of 0.69 (95% UI: 0.52–0.86), 0.90 (95% UI: 0.75–1.06), 1.23 (95% UI: 1.06–1.39), and 1.43 (95% UI: 1.28–1.58). Central Sub-Saharan Africa (CSA) showed the largest decrease in prevalence in the 2–4 and 5–14 years age groups, with EAPCs of −1.05 (95% UI: −1.14 to −0.95) and −0.95 (95% UI: −1.09 to −0.80), respectively. In contrast, the largest declines in prevalence among the 15–19 and 20–54 years age groups were observed in East Asia, with EAPCs of −1.45 (95% UI: −1.84 to −1.06) and −2.51 (95% UI: −3.11 to −1.91), respectively (see Supplementary Table S7).

**Figure 3:**
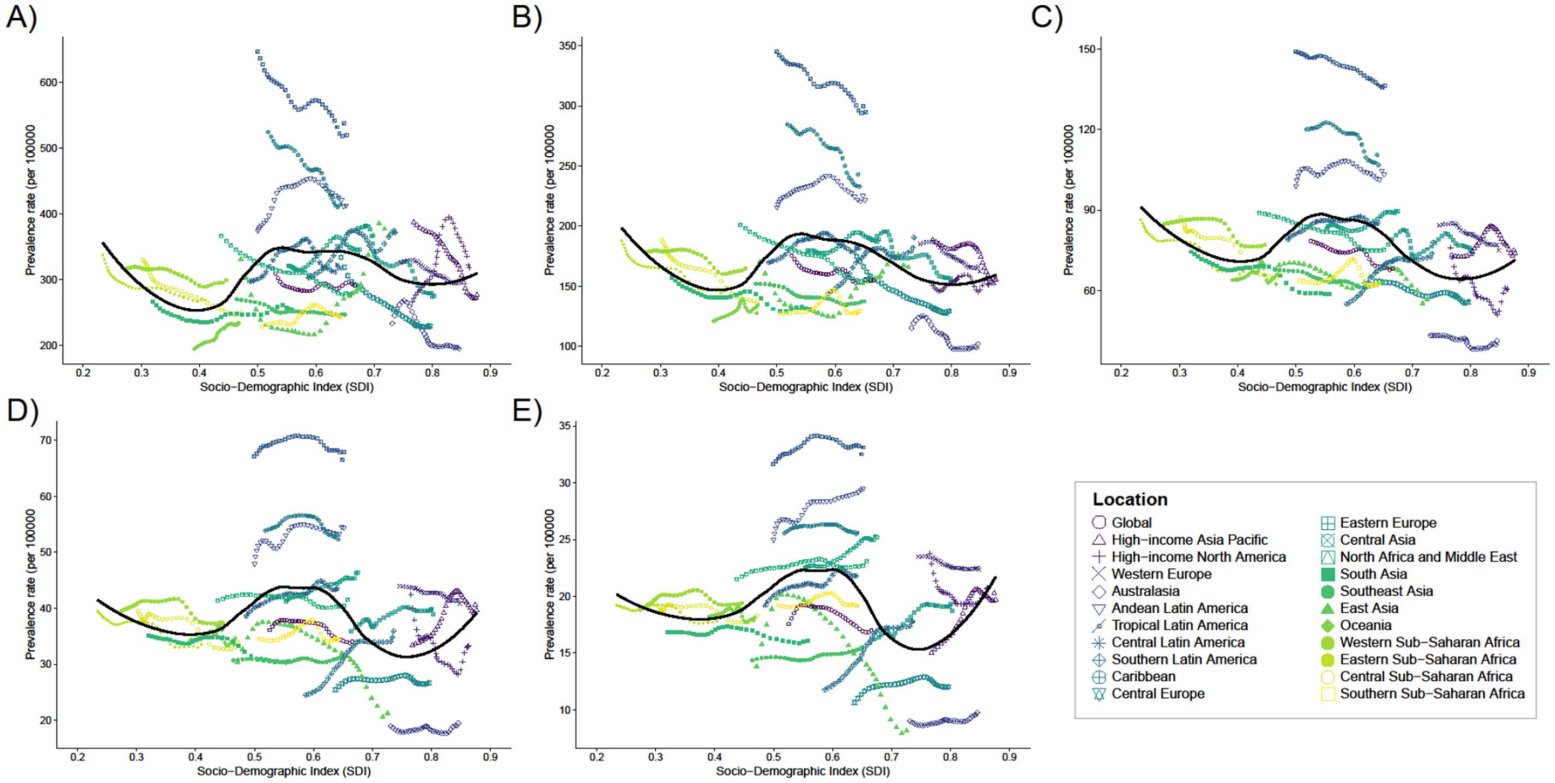
Trends in the prevalence of 21 regions with SDI for five different age groups, **(A)** <1 year old; **(B)** 2–4 years old; **(C)** 5–14 years old; **(D)** 15–19 years old; **(E)** 20–54 years old, from 1990 to 2021.

In 2021, the global mean prevalence of Digestive Congenital Anomalies (DCA) among infants under 1 year was 291.07 (95% UI: 227.65–373.09), with 10 regions, including Andean Latin America and the Caribbean, having rates higher than the global average, and 11 regions, such as Australasia and Central Europe, having rates lower than the global average. For children aged 2–4 years, the global mean prevalence was 154.87 (95% UI: 120.1–195.17), with 11 regions above the global average and 10 regions below. For children aged 5–14 years, the global mean prevalence was 68.21 (95% UI: 52.2–87.42), with 11 regions above the global average and 10 regions below. Adolescents aged 15–19 years had a global mean prevalence of 34.07 (95% UI: 25.65–43.13), with 13 regions above the global average and 8 regions below. For adults aged 20–54 years, the global mean prevalence was 17.01 (95% UI: 13.36–21.64), with 16 regions above the global average and five regions below (see Figure 3).

#### 3.3.2 Mortality

In 2021, the highest number of deaths from Digestive Congenital Anomalies (DCA) among individuals under 1 year, 2–4 years, and 5–14 years were all reported in Western Sub-Saharan Africa, with 10,384 deaths (95% uncertainty interval [UI]: 6,826–14,540), 1,014 deaths (95% UI: 683–1,419), and 1,014 deaths (95% UI: 683–1,419), respectively. The highest number of deaths among individuals aged 15–19 and 20–54 years were reported in South Asia, with 32 deaths (95% UI: 20–54) and 151 deaths (95% UI: 77–253), respectively. The number of deaths was close to zero across all four age groups, except for infants under 1 year, where the lowest number of deaths was reported in Australasia (22 deaths, 95% UI: 17–33).

Mortality rates for infants under 1 year showed largely negative EAPCs across regions, with the smallest decline in Central Latin America (EAPC: –0.09, 95% UI: −0.22 to 0.05) and the largest decline in Eastern Europe (EAPC: –4.83, 95% UI). Among children aged 2–4 years, the smallest decline in mortality was in Southern Latin America (EAPC: –0.3, 95% UI: −0.78 to 0.19), while the largest decline was in East Asia (EAPC: –6.31, 95% UI: −7.01 to −5.61). Among children aged 5–14 years, the largest increase in mortality was observed in Tropical Latin America (EAPC: 1.37, 95% UI: 0.91–1.82), while the largest decline was in High-income Asia Pacific (EAPC: –3.64, 95% UI: −4.16 to −3.12). In the 15–19 years age group, the largest increase was also observed in Tropical Latin America (EAPC: 3.15, 95% UI: 2.81–3.5), while the largest decline was in Australasia (EAPC: –3.65, 95% UI: −3.95 to −3.35). In the 20–54 years age group, the largest increase was observed in Tropical Latin America (EAPC: 2.0, 95% UI: 1.78–2.22), while the largest decline remained in Australasia (EAPC: –2.33, 95% UI: −2.57 to −2.10) (see Supplementary Table S7).

**Figure 4:**
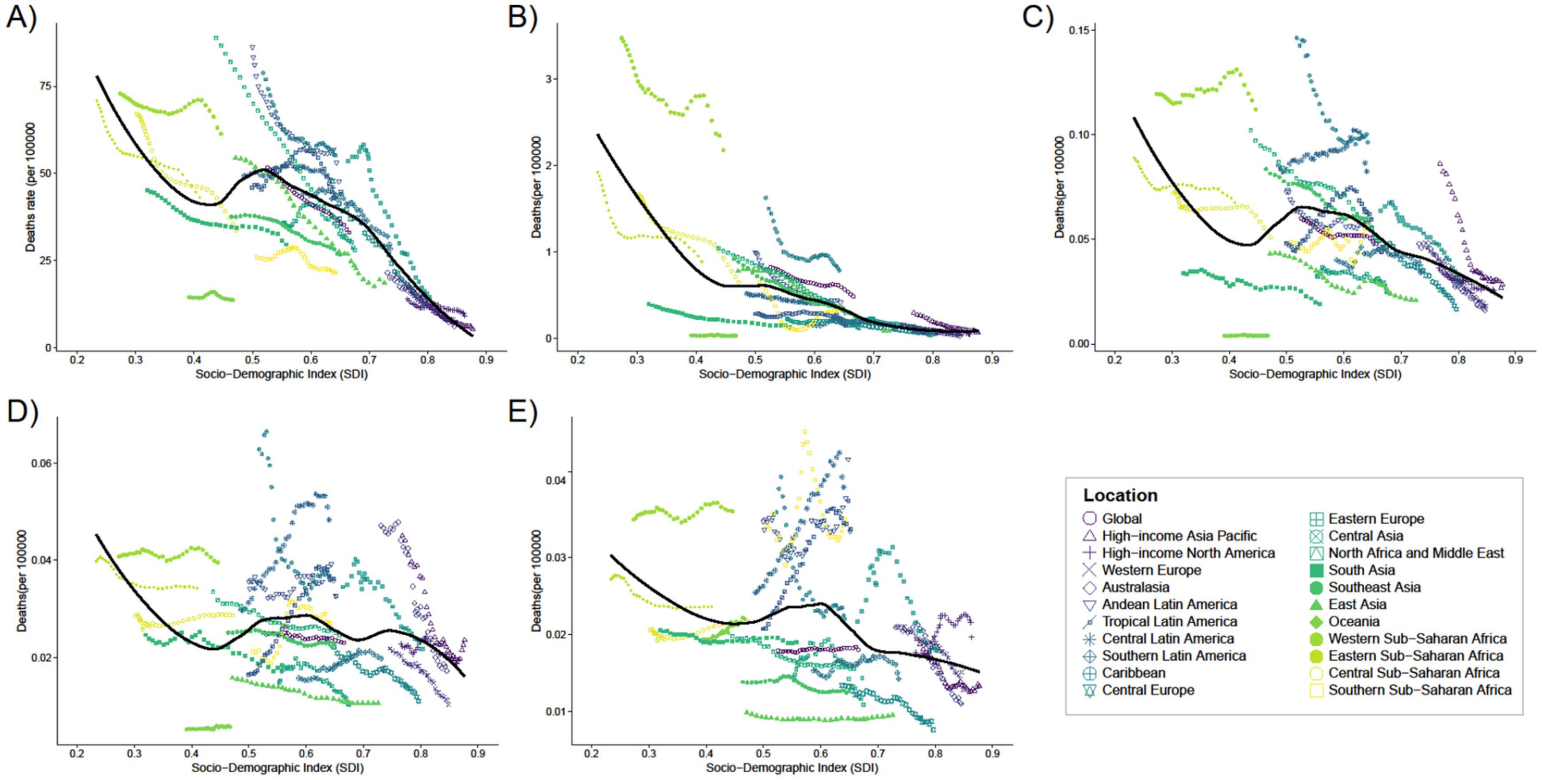
Trends in mortality with SDI for 21 Regions for five different age groups, **(A)** < 1 year old; **(B)** 2–4 years old; **(C)** 5–14 years old; **(D)** 15–19 years old; **(E)** 20–54 years old, from 1990 to 2021.

In 2021, the global average mortality rate for infants under 1 year was 33.03 (95% UI: 24.83–41.75), with seven regions, including Andean Latin America and the Caribbean, having mortality rates higher than the global average, and 14 regions, such as Australasia and Central Asia, having rates below the global average (Supplementary Table S7). Seven of these regions have mortality rates above the global average (e.g., Andean Latin America and Caribbean), while 14 regions have mortality rates below the global average (e.g., Australasia and Central Asia). The global average mortality rate for the remaining four age groups was close to zero (see Figure 4).

#### 3.3.3 DALYs

**Figure 5:**
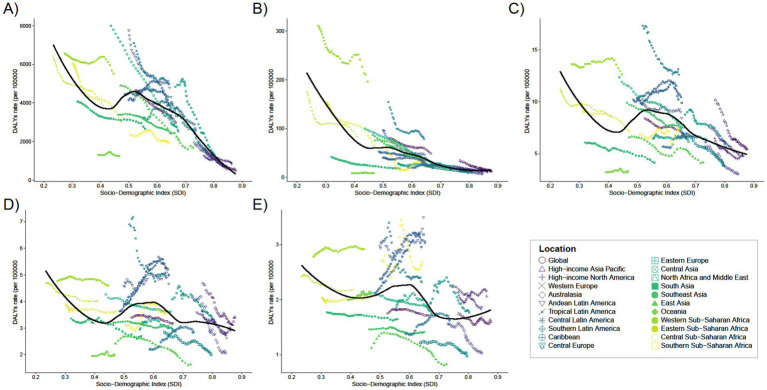
Trends in the rates of DALYs with SDI from 1990 to 2021 for 21 regions of five different age groups, **(A)** <1 year old; **(B)** 2–4 years old; **(C)** 5–14 years old; **(D)** 15–19 years old; **(E)** 20–54 years old.

**Figure 6:**
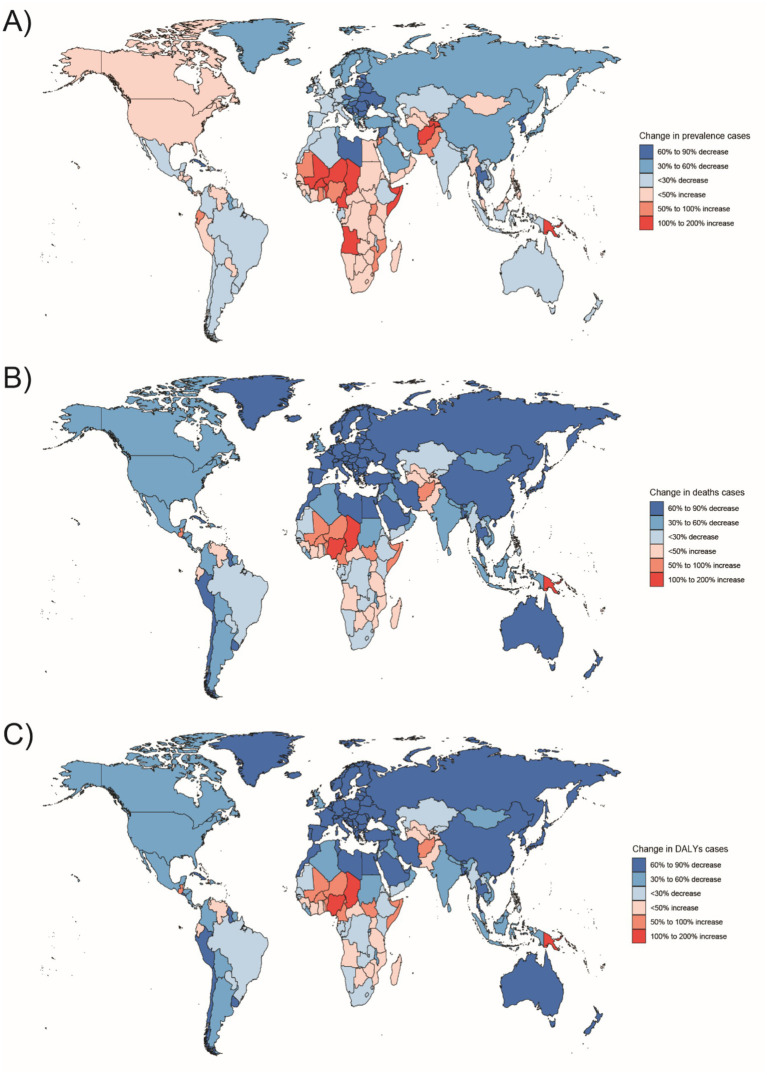
Changes in the prevalence of congenital malformations of the gastrointestinal tract (GI), deaths, and DALYs among people <1 year of age in 204 countries between 1990 and 2021, **(A)** Prevalence cases; **(B)** Deaths cases; **(C)** DALYs.

**Figure 7:**
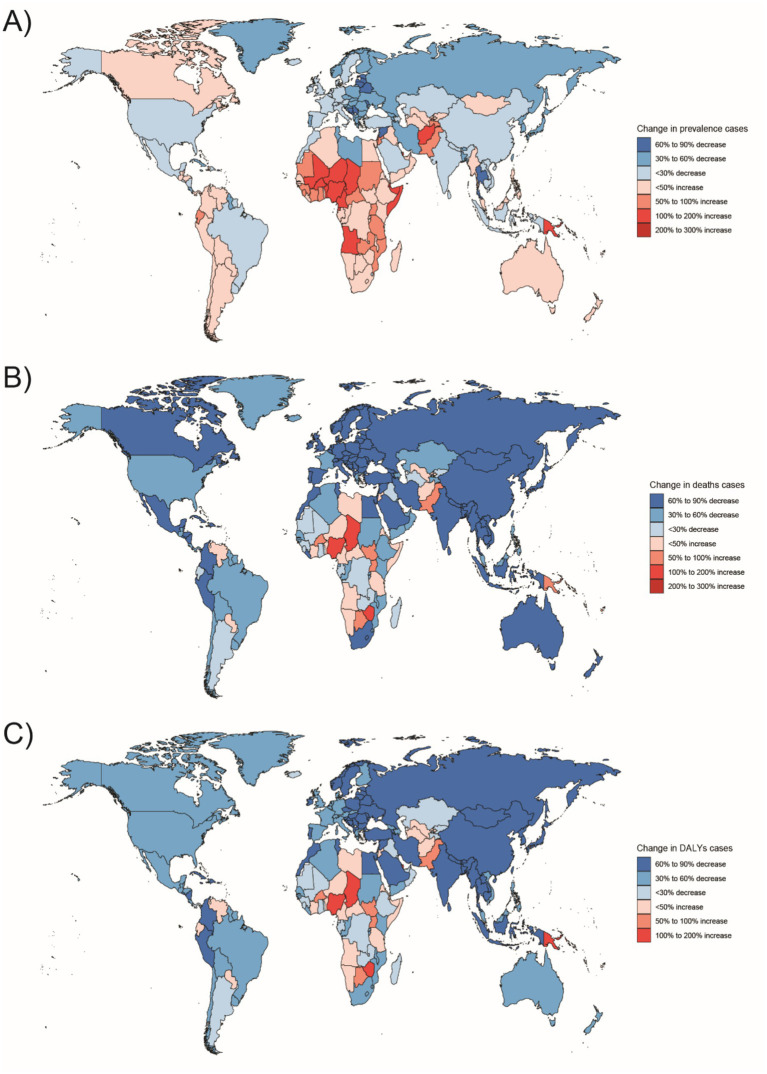
Changes in the prevalence of congenital malformations of the digestive tract, deaths, and DALYs among 2–4 year olds in 204 countries between 1990 and 2021, **(A)** Prevalence cases; **(B)** Deaths cases; **(C)** DALYs cases.

**Figure 8:**
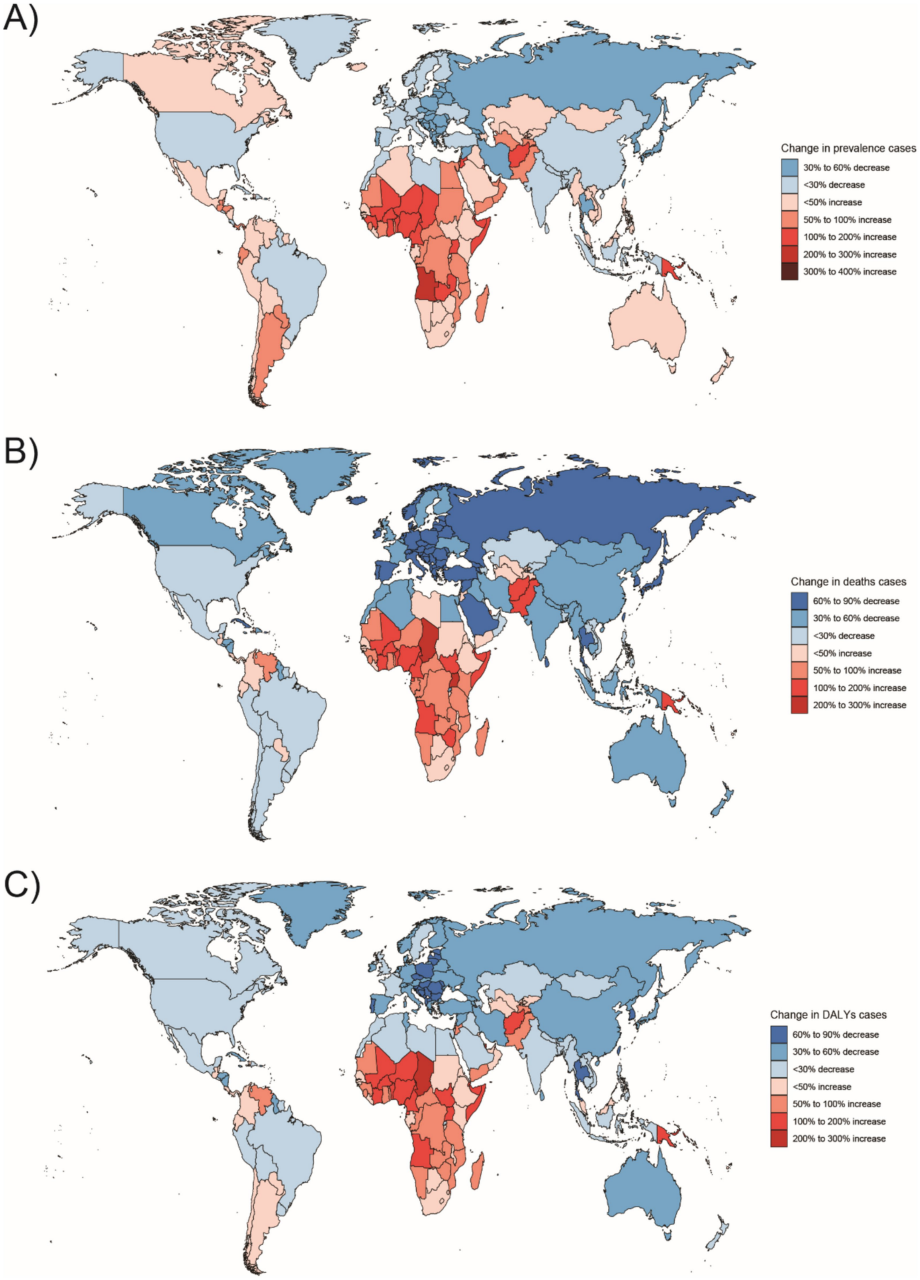
Changes in the prevalence of congenital malformations of the digestive tract, deaths, and DALYs among 5–14 year olds in 204 countries between 1990 and 2021, **(A)** Prevalence cases; **(B)** Deaths cases; **(C)** DALYs.

**Figure 9:**
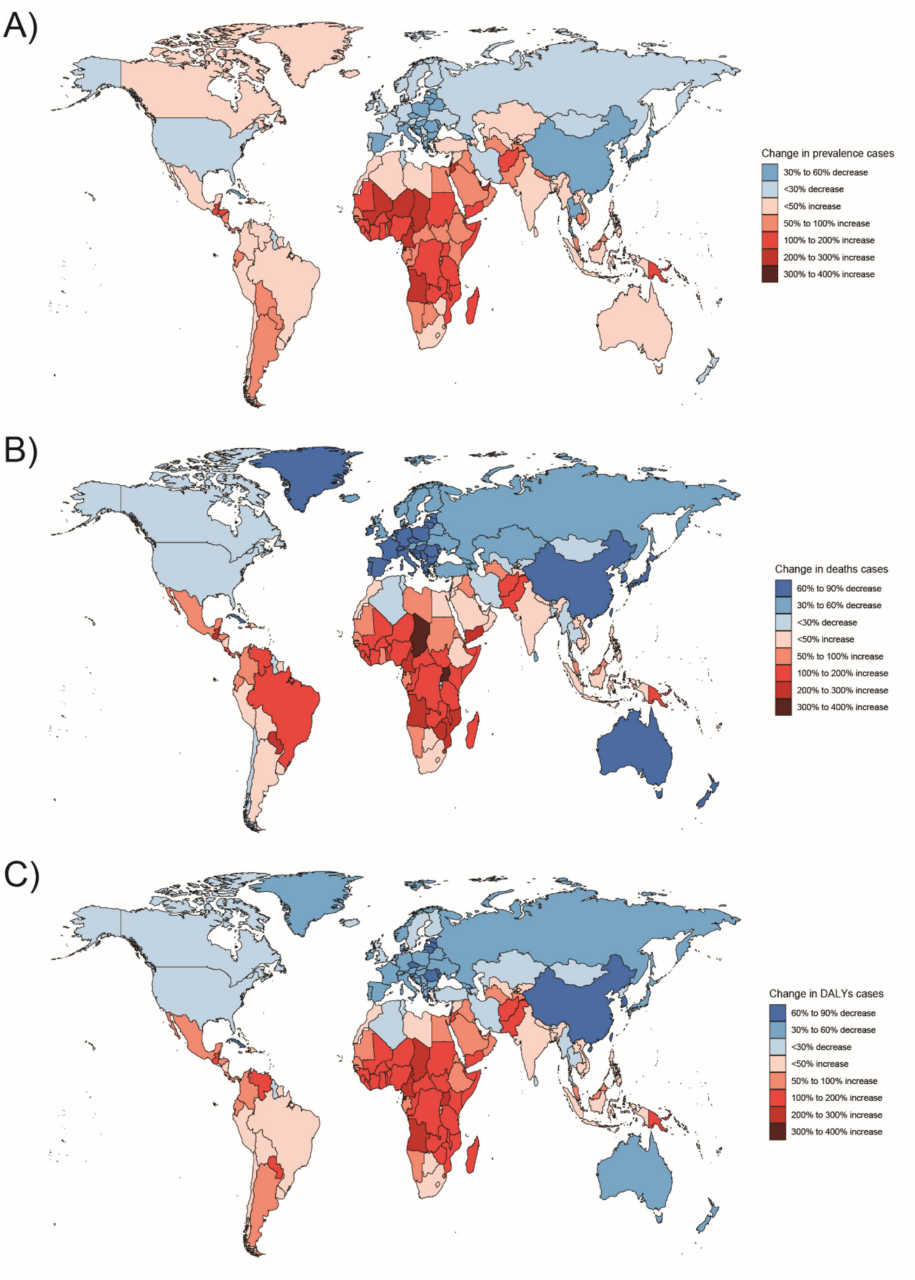
Changes in the prevalence of congenital malformations of the digestive tract, deaths, and DALYs among 15–19 year olds in 204 countries between 1990 and 2021, **(A)** Prevalence cases; **(B)** Deaths cases; **(C)** DALYs cases.

**Figure 10:**
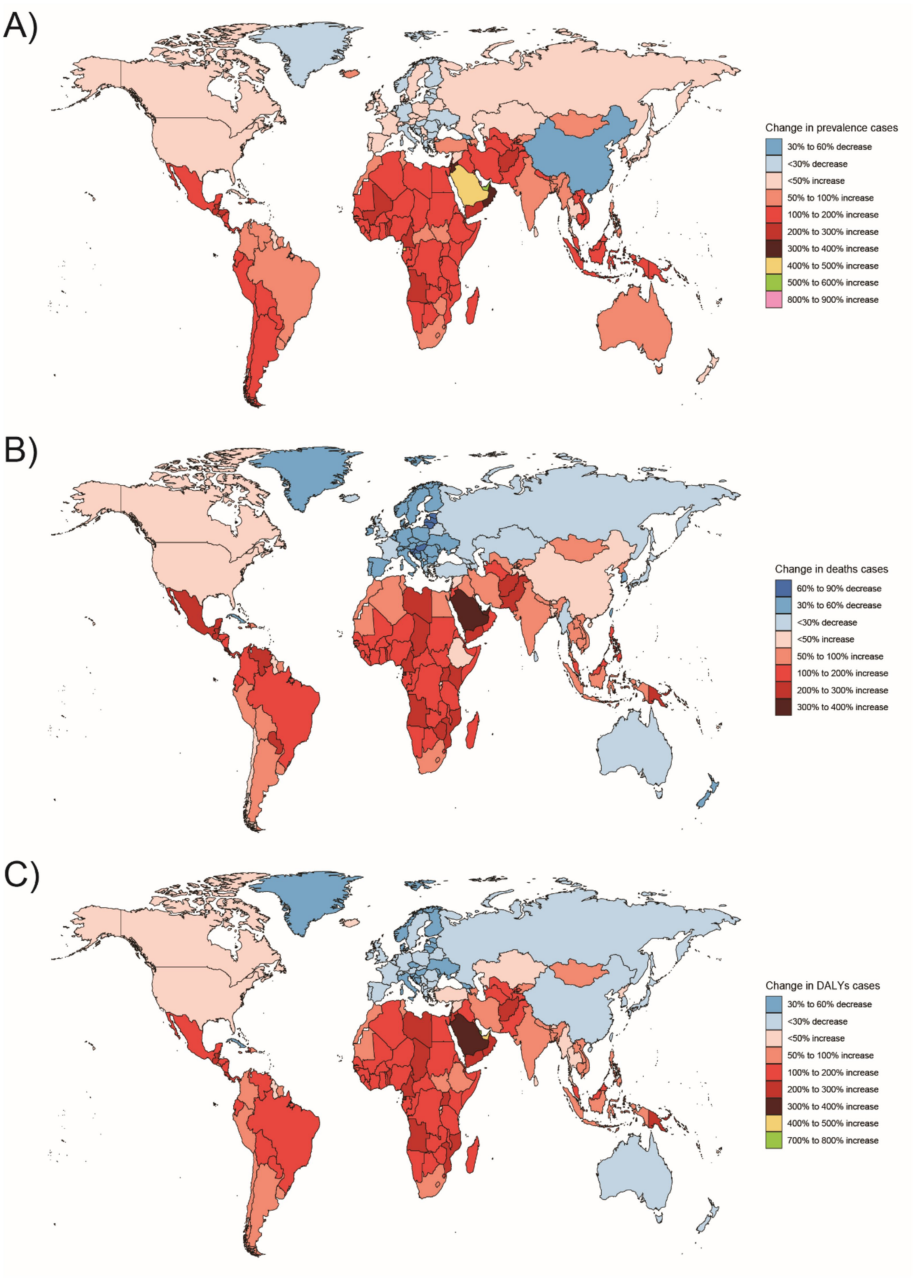
Changes in the prevalence of congenital malformations of the GI tract, deaths, and DALYs in 204 countries between 1990 and 2021, **(A)** Prevalence cases; **(B)** Deaths cases; **(C)** DALYs.

In 2021, the highest number of Digestive Congenital Anomalies (DCA)-related Disability Adjusted Life Years (DALYs) for people under 1 year, 2–4 years, and 5–14 years were all reported in Western Sub-Saharan Africa (WSSA), with 935,283 DALYs (95% uncertainty interval [UI]: 615,151–1,000,000). The highest number of DALYs for the 15–19 and 20–54 years age groups were also in Western Sub-Saharan Africa, with 91,722 DALYs (95% UI: 19,309–193,016) and 17,027 DALYs (95% UI: 10,843–25,692), respectively. South Asia had the highest number of DALYs in the population, with 4,758 DALYs (95% UI: 3,313–6,991) and 15,033 DALYs (95% UI: 10,697–21,692) in the 15–19 and 20–54 years age groups, respectively. Australasia had the lowest number of DALYs for infants under 1 year and children aged 2–4 years, with 1,990 DALYs (95% UI: 1,521–3,021) and 78 DALYs (95% UI: 52–117), respectively. The regions with the lowest DALYs for children aged 5–14, adolescents aged 15–19, and adults aged 20–54 years were in Oceania, with 106 DALYs (95% UI: 59–174), 26 DALYs (95% UI: 16–45), and 135 DALYs (95% UI: 68–214), respectively (Figures 5–10).

In Western Sub-Saharan Africa, these high DALY and death counts likely reflect a combination of contextual factors: protracted conflict and population displacement that disrupt antenatal care and neonatal referral, long travel distances with limited neonatal transport, shortages of pediatric surgical/an aesthesia workforce, oxygen and NICU capacity, high fertility yielding larger birth cohorts, and care-seeking delays and cultural stigma around congenital conditions—all compounded by under-registration of early neonatal deaths. In South Asia, urban–rural inequities, substantial out-of-pocket expenditure, variable antenatal ultrasound coverage/quality, and congested referral pathways plausibly elevate live-birth DCA incidence and DALYs despite macro-level gains. At the country level, India’s progress coexists with rural access gaps in prenatal screening and early surgical intervention, whereas Nigeria faces high birth rates, fragmented referral networks, and security-related service disruptions in certain states. By contrast, very low burdens in Australasia are consistent with near-universal facility births, centralized pediatric surgical services, robust neonatal transport, and mature congenital anomaly registries.

The rate of DALYs among infants under 1 year was largely negative in terms of EAPC, with the smallest decrease observed in Central Latin America (EAPC: –0.09, 95% UI: −0.22 to 0.05) and the largest decrease in Eastern Europe (EAPC: –4.81, 95% UI: −5.25 to −4.37). For children aged 2–4 years, the largest increase was observed in Oceania (EAPC: 0.07, 95% UI: −0.09 to 0.23), while the largest decrease occurred in East Asia (EAPC: –4.9, 95% UI: −5.3 to −4.5). For children aged 5–14 years, the largest increase was observed in Southern Latin America (EAPC: 0.43, 95% UI: 0.21–0.65), while the largest decrease occurred in Australasia (EAPC: –2.24, 95% UI: −2.34 to −2.13). For the 15–19 years age group, the largest increase was observed in Central Latin America (EAPC: 1.5, 95% UI: 1.35–1.66), while the largest decrease occurred in Australasia (EAPC: –2.68, 95% UI: −2.89 to −2.46). For the 20–54 years age group, the largest increase was also observed in Central Latin America (EAPC: 1.36, 95% UI: 1.26–1.45), while the largest decrease was in Australasia (EAPC: –1.57, 95% UI: −1.72 to −1.42) (see Supplementary Table S8).

These EAPC patterns are consistent with context: sharp declines in East Asia and Australasia align with multi-decadal investments in antenatal screening, neonatal intensive care, and timely pediatric surgery, whereas modest declines or increases in parts of Central/Tropical Latin America may reflect a mix of surveillance maturation (greater ascertainment), uneven perinatal coverage, rapid urbanization with changing environmental exposures, and periodic health-system shocks. In WSSA, insecurity and fragile supply chains can blunt mortality gains even when coverage expands. Policy responses should therefore be region-specific: (i) secure access to high-quality antenatal ultrasound and designated delivery at surgical hubs; (ii) strengthen neonatal transport/NICU and pediatric anesthesia capacity; (iii) reduce care-seeking delays through community engagement and financial protection; and (iv) upgrade congenital-anomaly registries and link them to vital registration to separate true changes from ascertainment effects.

### 3.4 National trends (204 country)

#### 3.4.1 Prevalence

In 2021, India (Republic of India) had the highest number of cases of Digestive Congenital Anomalies (DCA) across all age groups among 204 countries. Specifically, the number of cases was 56,084 (95% uncertainty interval [UI]: 41,867–75,751) for infants under 1 year, 90,629 (95% UI: 67,553–118,607) for children aged 2–4 years, and 149,355 (95% UI: 112,718–195,418) for those aged 5–14 years. Among those aged 15–19 years and 20–54 years, the number of cases was 40,654 (95% UI: 29,318–52,880) and 112,526 (95% UI: 86,882–144,488), respectively.

In 2021, Turkmenistan had the highest prevalence rates for infants under 1 year and children aged 2–4 years, with 574.33 (95% UI: 423.87–735.61) and 295.7 (95% UI: 221.74–377.54), respectively. The Federative Republic of Brazil had the highest prevalence for children aged 5–14 years, with 136.6 (95% UI: 106.14–173.97). The highest prevalence among adolescents aged 15–19 years and adults aged 20–54 years was observed in the Plurinational State of Bolivia, with 71.02 (95% UI: 52.84–91.97) and 39.59 (95% UI: 31.05–50.52), respectively.

From 1990 to 2021, the Independent State of Papua New Guinea saw the largest increase in prevalence among infants under 1 year, with a 175% increase (95% UI: 133–220%). The State of Qatar had the largest increases in prevalence among children aged 2–4 years, 5–14 years, and adults aged 20–54 years, with increases of 209% (95% UI: 154–266%), 308% (95% UI: 239–395%), and 875% (95% UI: 727%–1,070%), respectively.

In terms of average annual changes in prevalence, the largest increase among infants under 1 year and children aged 2–4 years was observed in Turkmenistan, with EAPCs of 2.17 (95% UI: 1.9–2.44) and 1.81 (95% UI: 1.58–2.05), respectively. The largest decrease was in the Republic of Estonia, with EAPCs of −3.37 (95% UI: −3.56 to −3.17) and −2.37 (95% UI: −2.54 to −2.21), respectively. The largest increase in prevalence among children aged 5–14 years was also observed in Turkmenistan (EAPC: 1.34, 95% UI: 1.18–1.5), while the largest decrease occurred in the Kingdom of Norway. Among adolescents aged 15–19 years, the largest increase in prevalence was observed in the Republic of Chile (EAPC: 1.48, 95% UI: 1.35–1.62), while the largest decrease occurred in the People’s Republic of China (EAPC: –2.65, 95% UI: −3.28 to −2.01). For adults aged 20–54 years, the largest increase in prevalence was observed in the Eastern Republic of Uruguay (EAPC: 1.63, 95% UI: 1.44–1.83), while the largest decrease occurred in China (EAPC: –2.65, 95% UI: −3.28 to −2.01).

#### 3.4.2 Mortality

In 2021, India (Republic of India) had the highest number of deaths from Digestive Congenital Anomalies (DCA) among people under 1 year, aged 15–19 years, and aged 20–54 years across 204 countries, with 6,623 deaths (95% uncertainty interval [UI]: 3,725.41–11,359.72), 24.44 deaths (95% UI: 15.63–40.19), and 147.95 deaths (95% UI: 87.4–257.36), respectively. The Federal Republic of Nigeria had the highest number of deaths in the 2–4 and 5–14 years age groups, with 578 deaths (95% UI: 74.94–1,364.93) and 77.68 deaths (95% UI: 37.97–131.33), respectively.

In 2021, the country with the highest mortality rate for infants under 1 year was the Plurinational State of Bolivia, at 81.83 (95% UI: 51.62–114.44). The Republic of Mali had the highest mortality rate for children aged 2–4 years, at 2.67 (95% UI: 0.41–6.32) Mortality rates for other age groups were essentially zero in several countries.

From 1990 to 2021, the country with the largest increase in deaths among infants under 1 year and children aged 5–14 years was the Republic of Chad, with increases of 153% (95% UI: 57–370%) and 291% (95% UI: 104–778%), respectively. The country with the largest increase in deaths among children aged 2–4 years was Tokelau, with a 429% increase (95% UI: −32 to 2,703%). The Republic of Uganda saw the largest increase in deaths among adolescents aged 15–19 years, with a 317% increase (95% UI: 90–1,000%). The Kingdom of Saudi Arabia had the largest increase in deaths among adults aged 20–54 years, with a 363% increase (95% UI: 159–757%). (see Supplementary Table S11 and Supplementary Figures 1B–5B).

Among infants under 1 year, the Republic of Guatemala had the largest increase in mortality (EAPC: 4.22, 95% UI: 3.42–5.02), while the Northern Mariana Islands had the largest decrease (EAPC: –8.45, 95% UI: −9.8 to −7.1). Among children aged 2–4 years, the Republic of Zimbabwe had the largest increase in mortality (EAPC: 6.78, 95% UI: 4.43–9.19), while the Republic of Serbia had the largest decrease (EAPC: –7.58, 95% UI: −8.36 to −6.79). Among children aged 5–14 years, the largest increase in mortality was observed in Zimbabwe (EAPC: 3.79, 95% UI: 2.98–4.6), while the largest decrease occurred in the Northern Mariana Islands (EAPC: –6.28, 95% UI: −7.46 to −5.09). Among adolescents aged 15–19 years, the Bolivarian Republic of Venezuela had the largest increase in mortality (EAPC: 4.06, 95% UI: 3.68–4.44), while the Northern Mariana Islands had the largest decrease (EAPC: –5.49, 95% UI: −8.86 to −1.99). Among adults aged 20–54 years, the Kingdom of Lesotho had the largest increase in mortality (EAPC: 3.15, 95% UI: 2.61–3.69), while the Kingdom of Norway had the largest decrease (EAPC: –4.49, 95% UI: −5.1 to −3.88).

In 2021, the global average mortality rate for infants under 1 year was 33.03 (95% UI: 24.83–41.75), with 66 countries reporting mortality rates above the global average and 138 countries below the global average. Mortality rates for the other four age groups were close to zero (see Supplementary Figures 11B–15B).

#### 3.4.3 DALYs

In 2021, India (Republic of India) had the highest number of Disability-Adjusted Life Years (DALYs) related to Digestive Congenital Anomalies (DCA) among people under 1 year, and those aged 5–14, 15–19, and 20–54 years across 204 countries, with 598,187.55 DALYs (95% uncertainty interval [UI]: 337,709.85–1,023,954.78), 9,718.06 DALYs (95% UI: 6,887.86–13,819.51), 3,583.38 DALYs (95% UI: 2,517.54–5,191.07), and 12,142.65 DALYs (95% UI: 8,667.46–17,880.97), respectively. The country with the highest number of DALYs among children aged 2–4 years was the Federal Republic of Nigeria, with 51,749.34 DALYs (95% UI: 8,160.17–119,873.27).

In 2021, the country with the highest rate of Disability-Adjusted Life Years (DALYs) among infants under 1 year was the Plurinational State of Bolivia, at 7,379.33 (95% UI: 4,661.58–10,308.45). The countries with the highest rates of DALYs among children aged 2–4 years, 5–14 years, and adults aged 20–54 years were the Republic of Mali, with rates of 239.5 (95% UI: 42.35–555.16), 16.9 (95% UI: 7.59–47.5), and 4.13 (95% UI: 1.39–13.04), respectively. Among adolescents aged 15–19 years, the Bolivarian Republic of Venezuela had the highest rate of DALYs, at 9.05 (95% UI: 5.76–12.72).

From 1990 to 2021, the largest increase in the number of Disability-Adjusted Life Years (DALYs) among infants under 1 year, children aged 2–4 years, and those aged 5–14 years occurred in the Republic of Chad, with increases of 153% (95% UI: 57–369%), 164% (95% UI: 28–920%), and 258% (95% UI: 128–511%), respectively. Among adolescents aged 15–19 years, the Republic of Equatorial Guinea had the largest increase in the number of DALYs, with a 333% increase (95% UI: 155–710%). Among adults aged 20–54 years, the State of Qatar had the largest increase in the number of DALYs, with a 746% increase (95% UI: 580–944%). (see Supplementary Table S12 and Supplementary Figures 1C–5C).

In terms of mean annual change in DALYs rates, among infants under 1 year, the Republic of Guatemala had the largest increase (EAPC: 4.2, 95% UI: 3.4–5.0), while the Northern Mariana Islands had the largest decrease. Among children aged 2–4 years, the Republic of Zimbabwe had the largest increase in DALYs (EAPC: 4.35, 95% UI: 3.0–5.72), while the Democratic Republic of Sao Tome and Principe had the largest decrease (EAPC: –6.69, 95% UI: −7.45 to −5.92). Venezuela had the largest increase in DALYs rates among children aged 5–14 years and adults aged 20–54 years (EAPC: 1.78, 95% UI: 1.59–1.97, and 2.15, 95% UI: 2.01–2.3, respectively). The Kingdom of Norway had the largest decrease (EAPC: –3.58, 95% UI: −3.9 to −3.27, and −2.6, 95% UI: −2.92 to −2.27, respectively). Among adolescents aged 15–19 years, Venezuela had the largest increase in DALYs rates (EAPC: 3.19, 95% UI: 2.92–3.46), while Australia had the largest decrease (EAPC: –2.73, 95% UI: −2.99 to −2.46). (see Supplementary Table S12 and Supplementary Figures 6C–10C).

In 2021, the global mean Disability-Adjusted Life Years (DALYs) rate for infants under 1 year was 2,981.89 (95% UI: 2,246.46–3,768.14), with 66 countries having rates above the global average and 138 countries below. The global mean DALYs rate for children aged 2–4 years was 49.13 (95% UI: 22.61–83.91), with 47 countries above the global average and 157 countries below. The global mean DALYs rate for children aged 5–14 years was 6.6 (95% UI: 4.95–8.81), with 99 countries above the global average and 105 countries below. For adolescents aged 15–19 years, the global mean DALYs rate was 3.18 (95% UI: 2.33–4.36), with 110 countries above the global average and 94 countries below. The global mean DALYs rate for adults aged 20–54 years was 1.71 (95% UI: 1.29–2.26), with 130 countries above the global average and 74 countries below (see Supplementary Figures 11C–15C).

## 4 Future trends

**Figure 11:**
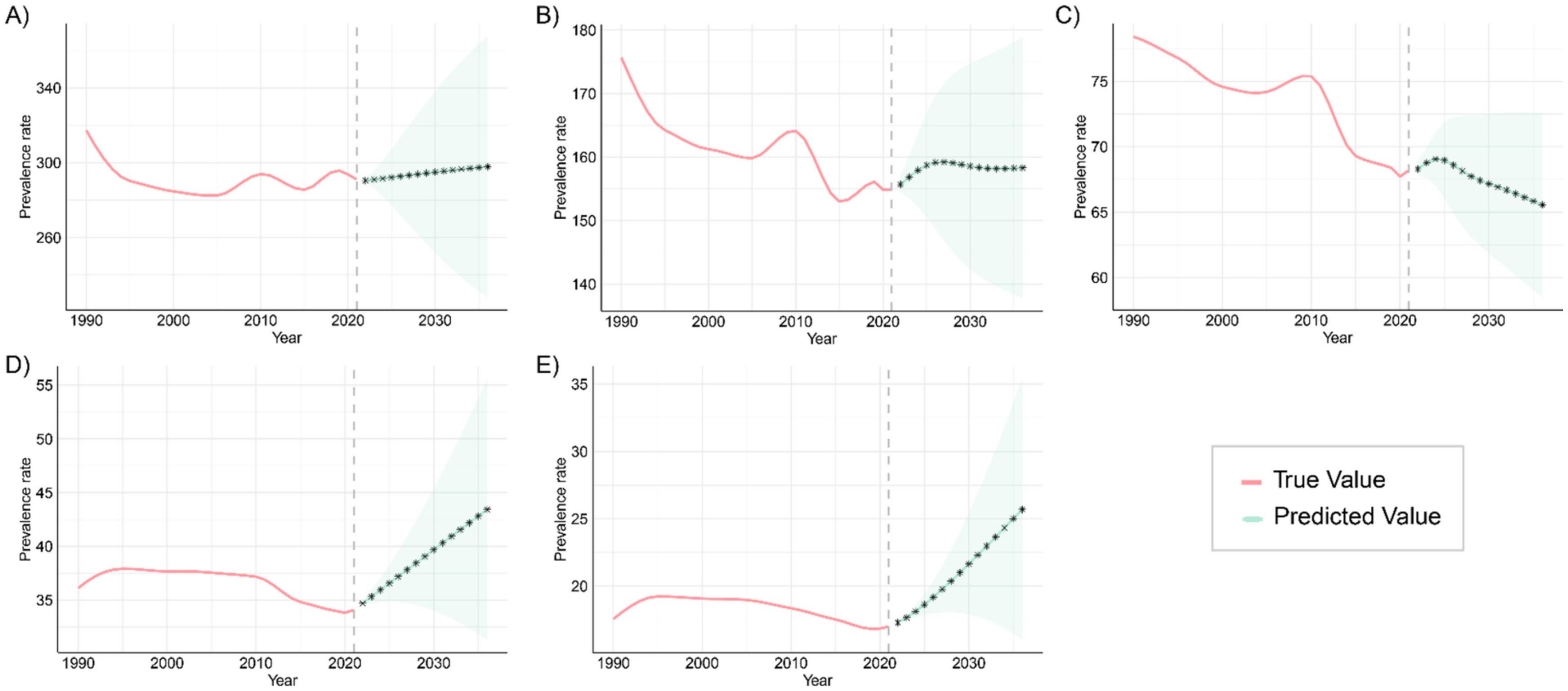
Projected trends in the prevalence of congenital malformations of the GI tract globally over the next 15 years (2021–2046), with the red and blue lines representing true and projected prevalence values, respectively, and the blue shaded area indicating the 95% PI for **(A)** <1 year old; **(B)** 2–4 years old; **(C)** 5–14 years old; **(D)** 15–19 years old; **(E)** 20–54 years old.

**Figure 12:**
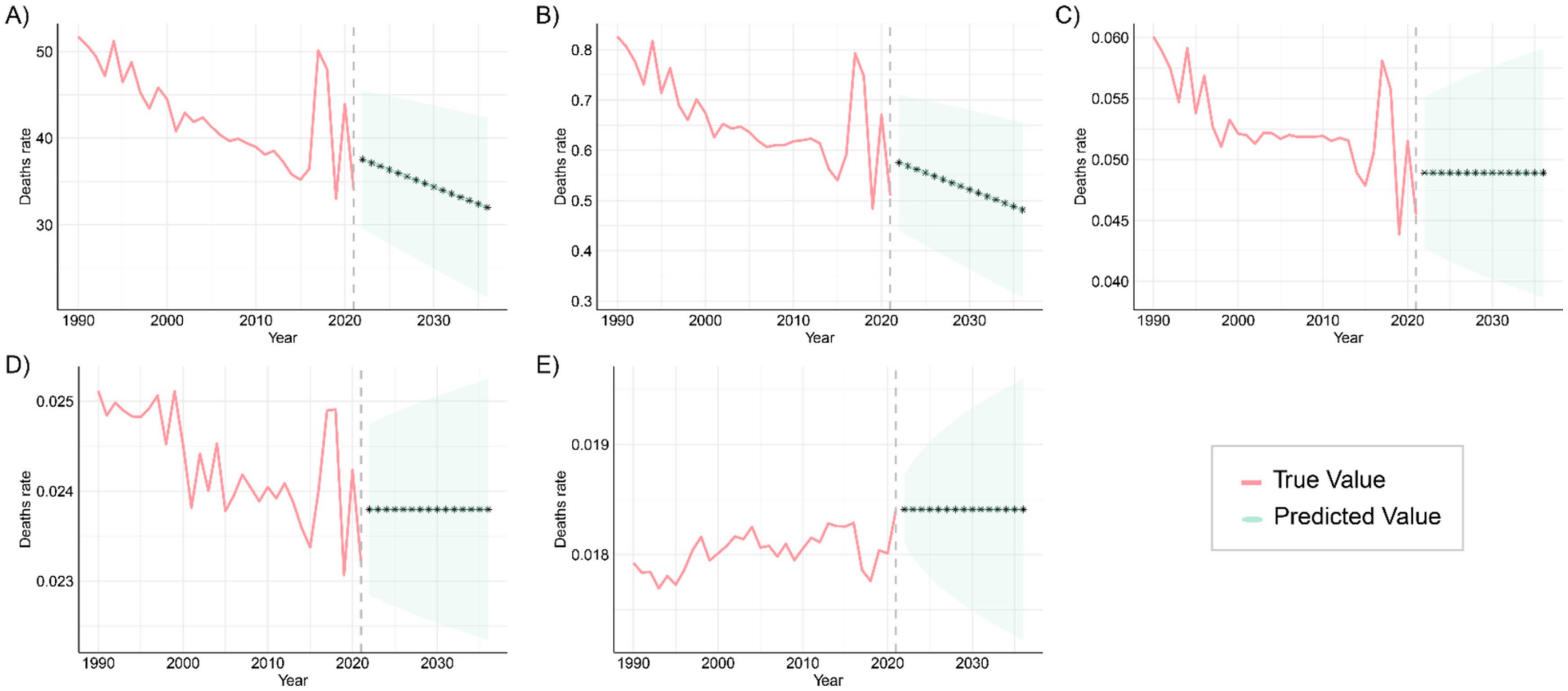
Predicted trends in global mortality from congenital malformations of the digestive tract over the next 15 years (2021–2046), with the red and blue lines representing true and predicted values of mortality, respectively, and the blue shaded area indicating the 95% PI, **(A)** <1 year old; **(B)** 2–4 years old; **(C)** 5–14 years old; **(D)** 15–19 years old; **(E)** 20–54 years old.

The ARIMA model was applied to quantitatively assess trends in the prevalence and mortality of gastrointestinal (GI) congenital malformations over the next 15 years. As shown in Figure 11, the prevalence among infants under 1 year, adolescents aged 15–19 years, and adults aged 20–54 years is projected to continue increasing over the next 15 years, while the prevalence among children aged 2–4 years and 5–14 years is expected to fluctuate, but without a significant increase. The projected mortality trends in Figure 12 suggest that mortality rates for infants under 1 year and children aged 2–4 years will steadily decline over the next 15 years, while mortality rates for the other three age groups are expected to remain unchanged. For each age–outcome series, we fit non-seasonal ARIMA models (annual frequency) using auto.arima (AICc selection). The final ARIMA(p,d,q) orders and information criteria (AICc/BIC) for all series used in Figures 11, 12 are reported in Table 2; residual diagnostics (Ljung–Box) indicated no remaining autocorrelation.

**Table 2:** ARIMA model specifications (p, d, q) and fit statistics (AICc, BIC) for age-stratified mortality (“Deaths”) and prevalence of digestive congenital anomalies (Global, GBD 2021, 1990–2021).

Measure	Age	p	d	q	AICc	BIC
Deaths	<1 year	1	1	0	−17.847904	−14.43483
Deaths	2–4 years	1	1	0	−231.32088	−227.90781
Deaths	5–14 years	1	1	0	−377.58295	−374.16988
Deaths	15–19 years	0	1	0	−433.25817	−430.8188
Deaths	20–54 years	0	1	0	−477.3762	−476.0802
Prevalence	<1 year	2	0	2	88.297628	93.73204
Prevalence	2–4 years	2	1	0	66.73735	70.15042
Prevalence	5–14 years	2	1	2	22.48467	27.58859
Prevalence	15–19 years	0	2	1	−49.60205	−47.2441
Prevalence	20–54 years	1	2	1	−126.5089	−123.2284

These patterns are plausibly driven by a cohort-survival effect (more children with DCA surviving into adulthood), gaps in transition of care and limited adult congenital services leading to late complications and re-interventions, and improvements in adult-care diagnosis/coding and registry maturation that increase case ascertainment; conversely, the continuing decline in infant and early-child mortality aligns with sustained gains in perinatal and neonatal care, and the fluctuations at ages 2–4 and 5–14 likely reflect heterogeneous progress in referral pathways, diagnostic capacity, and registry coverage across regions. From a planning perspective, the projections imply scaling high-quality antenatal ultrasound and designating delivery at surgical hubs; strengthening neonatal transport, NICU, pediatric anesthesia, and timely access to pediatric surgery—especially in low-SDI settings; establishing structured child-to-adult transition clinics and expanding adult congenital DCA services to manage late sequelae; and enhancing congenital-anomaly registries and linking them to vital registration to distinguish true changes from ascertainment effects, with priority to high-burden regions such as Western Sub-Saharan Africa and South Asia. Finally, these forecasts are autoregressive ARIMA projections displayed with 80%/95% prediction intervals that widen with horizon; therefore, we emphasize near-term (≈5–10 years) planning utility and treat the outer horizon as scenario-level and contingent on structural changes (e.g., screening expansion, coding transitions, or system shocks).

## 5 Discussion

This study provides a comprehensive analysis of global trends in the prevalence, mortality, and disability-adjusted life years (DALYs) associated with digestive congenital anomalies (DCA) across different age groups, sociodemographic index (SDI) regions, and countries, based on Global Burden of Disease (GBD) 2021 data. The study also employs an ARIMA model to project trends for the next 15 years.

Globally, the burden of digestive congenital anomalies (DCA) in infants under 1 year is significantly higher than in other age groups, in terms of prevalence, mortality, and disability-adjusted life years (DALYs). This may be attributed to the vulnerability and physiological characteristics of infants at birth, including an underdeveloped digestive system (24), heightened sensitivity to structural malformations, and a narrower window for treatment. The greatest increase in DCA prevalence and mortality among infants under 1 year was observed in low-SDI regions, likely reflecting deficiencies in healthcare infrastructure, prenatal screening, and neonatal intensive care capacity in these areas. In addition, part of the apparent rise likely reflects improved case ascertainment as facility births, neonatal units, and referral pathways expand, although under-registration and misclassification persist. Declines in competing neonatal causes (e.g., sepsis, birth asphyxia) may also increase the number of infants who survive long enough to be diagnosed (“survival shift shifts”) (25). Changing risk profiles—including maternal age distribution, under-nutrition/micronutrient deficiency, diabetes/obesity, infections, and environmental exposures—and lower coverage/resolution of antenatal screening and barriers to pregnancy termination may further raise live-birth incidence in these settings. In low- and middle-income countries, in particular, newborns often lack access to adequate medical care, resulting in delayed diagnosis and intervention for DCA, which further increases mortality and the DALY burden. To address these issues, targeted interventions at both the policy and practice levels are required, such as improving prenatal diagnosis rates, expanding neonatal intensive care capacity, and enhancing neonatal emergency and surgical intervention skills (26). Interpretation of the observed temporal and cross-SDI differences should account for potential confounding from (i) improved prenatal detection and, where lawful, termination that lower live-birth DCA incidence in high-SDI settings, (ii) time-varying changes in the birth prevalence of DCA, and (iii) co-morbidities (e.g., prematurity, sepsis, congenital heart disease, malnutrition) that modify survival and DALYs; accordingly, absolute levels should be interpreted alongside the reported 95% uncertainty intervals (27).

In high SDI areas, the burden of DCA has significantly declined across all age groups, particularly in terms of mortality and DALYs. These regions benefit from advanced medical technologies, well-established prenatal screening systems, universal public health policies, and extensive health insurance coverage. Such resources ensure high-quality surgical care and comprehensive management, resulting in notable reductions in the burden of DCA. Moreover, broader antenatal screening and—where lawful—access to pregnancy termination likely reduce live-birth incidence in high-SDI settings, accentuating the contrast with low-SDI regions. The experience of high-income countries shows that early intervention, standardized surgical treatment protocols, and long-term care effectively reduce mortality and improve quality of life in DCA patients (28). Implementing these strategies in low-SDI areas could significantly reduce disease burden and global health inequalities (29).

At the national level, countries with large populations, such as India and Nigeria, report the highest number of DCA cases and DALYs across most age groups, highlighting the significant disease burden in these regions. Despite improvements in India’s healthcare system in recent decades, limited healthcare access in rural and underdeveloped areas, especially in prenatal screening and early intervention, has slowed progress in managing congenital anomalies like DCA. Similarly, Nigeria faces a high disease burden due to limited healthcare resources, poor infrastructure, and high birth rates. Addressing these challenges requires sustained governmental and international investment, including expanding basic healthcare infrastructure, improving healthcare accessibility in remote areas, and raising awareness of health education and disease prevention at the community level (30, 31).

Additionally, countries like Bolivia and Mali exhibit the highest global DALY rates across multiple age groups, indicating severe deficiencies in managing the DCA disease burden. This may be linked to factors such as socioeconomic inequality, fragile healthcare systems, and inadequate implementation of health policies (32). Strengthening healthcare in these regions, particularly through improved antenatal diagnosis and enhanced neonatal care and surgical treatment capacity, will be crucial for reducing their DALY burden.

Projections of future trends indicate the potential direction of change in the global DCA burden. ARIMA model forecasts suggest that DCA prevalence will continue to rise over the next 15 years among those <1 year old, 15–19 years old, and 20–54 years old. This trend may be driven by factors like population growth, rising birth rates, and unequal access to healthcare in certain regions. Low- and middle-income countries will face increasing DCA cases, further straining their limited healthcare resources and infrastructure. While fluctuating increases in DCA prevalence are expected in the 2–4 and 5–14 age groups, this underscores the need to strengthen prevention and early intervention for these populations. Future strategies should focus on optimizing prenatal screening and neonatal care to alleviate the disease burden through effective interventions and technologies. For adults (20–54 years), part of the observed increase is plausibly a cohort-survival effect as more children with DCA now live into adulthood. In parallel, gaps in transition of care and limited adult congenital services can lead to late complications and re-interventions, increasing both prevalence and case ascertainment. In several settings, diagnostic/coding improvements and registry maturation in adult services may also raise recorded prevalence even without proportional changes in incident malformations (33).

Mortality projections indicate a continued decline in the <1 year and 2–4 years age groups, likely due to global improvements in neonatal and infant care, including advancements in preterm management, enhanced neonatal monitoring, and resuscitation techniques (34, 35). However, the stabilization of mortality rates in the 5–14, 15–19, and 20–54 age groups suggests that current interventions have limited impact on these populations. Future efforts should focus on more precise and personalized interventions targeting the specific risk factors and pathological characteristics of these age groups to improve management outcomes and reduce the long-term disease burden. Among adults, persistent mortality and DALYs likely reflect unaddressed long-term sequelae (e.g., obstruction, strictures, nutritional compromise), the accumulation of comorbidities with age, and limited access to timely re-intervention and multidisciplinary follow-up. Establishing structured child-to-adult transition clinics, expanding adult congenital surgical capacity, and strengthening longitudinal registries could help mitigate this residual burden.

To translate these findings into action, we prioritize tailored measures rather than generic recommendations. In low-SDI settings—especially Western Sub-Saharan Africa and South Asia—set measurable targets: antenatal ultrasound coverage ≥80%, designated delivery at pediatric surgical hubs ≥70%, and median neonatal transfer time ≤6 h within 5 years, alongside baseline NICU and pediatric anesthesia capacity and financial protection for timely surgery. To address the emerging adult (20–54 y) burden, establish structured child-to-adult transition pathways and expand adult congenital DCA services, tracking retention, complication, and re-intervention rates. Finally, strengthen congenital-anomaly registries and linkage to vital registration to distinguish true changes from ascertainment and to monitor progress against these targets. Beyond epidemiologic trends, these findings carry an ethical imperative: the concentration of preventable DCA deaths and disability in low-SDI settings—driven by limited access to basic prenatal screening, neonatal intensive care, and pediatric surgical services—represents a health-equity failure that should be explicitly acknowledged and prioritized in policy and financing decisions.

This study underscores the need to enhance maternal and neonatal care services in low SDI regions, particularly by improving the accessibility and quality of care in rural and remote areas. Middle-income countries should build on their existing healthcare investments, with a focus on disease management, early screening, and life-cycle care. The experience of high-income countries demonstrates that advanced medical technologies and comprehensive public health policies can substantially reduce the burden of DCA. Expanding these successful strategies globally will help alleviate the disease burden and address health inequalities. Future research should examine the socioeconomic impact of DCA, healthcare burdens at the household and community levels, and the role of environmental and genetic factors in DCA pathogenesis. Uncovering deeper disease mechanisms through interdisciplinary research will enable more precise prevention, control, and intervention strategies.

## Data Availability

The original contributions presented in the study are included in the article/Supplementary material, further inquiries can be directed to the corresponding author.
