# Supplementary material for: Global, regional, and national differences in the incidence and mortality of digestive congenital anomalies from 1990 to 2021, with projections for future trends
Source: Front Public Health. 2025 Oct 14;13:1640700. doi: 10.3389/fpubh.2025.1640700 (PMC12558871; doi:10.3389/fpubh.2025.1640700)
Supplement: Supplementary file 1 [file Data_Sheet_1.docx]

Supplementary Material

# Supplementary Figures

**
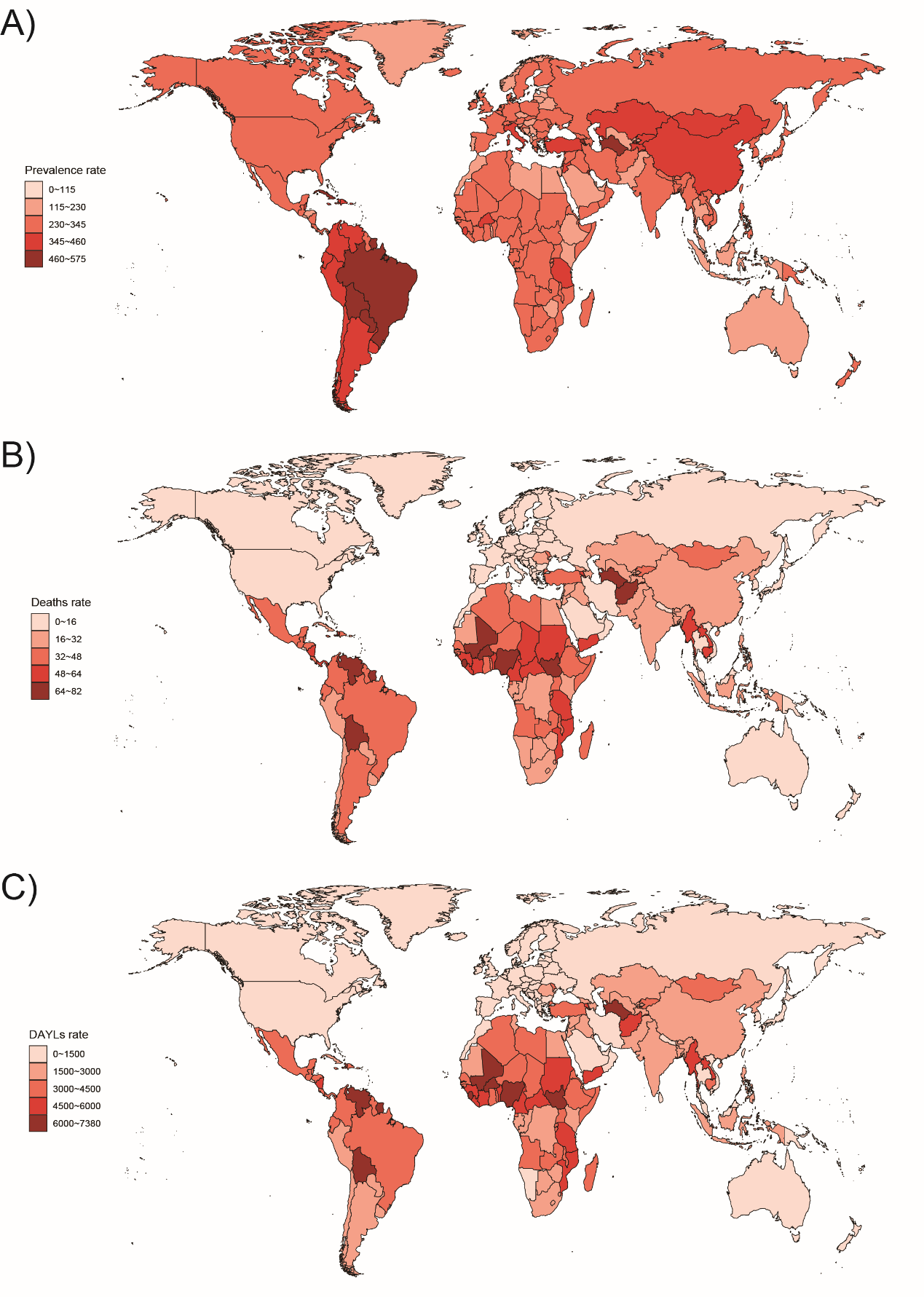
**

**Supplementary Figure 1. Trends in prevalence, mortality, and DALY rates for congenital gastrointestinal malformations in children under 1 year of age across 204 countries from 1990 to 2021. A) Prevalence rate; B) Mortality rate; C) DALY rate.**

**
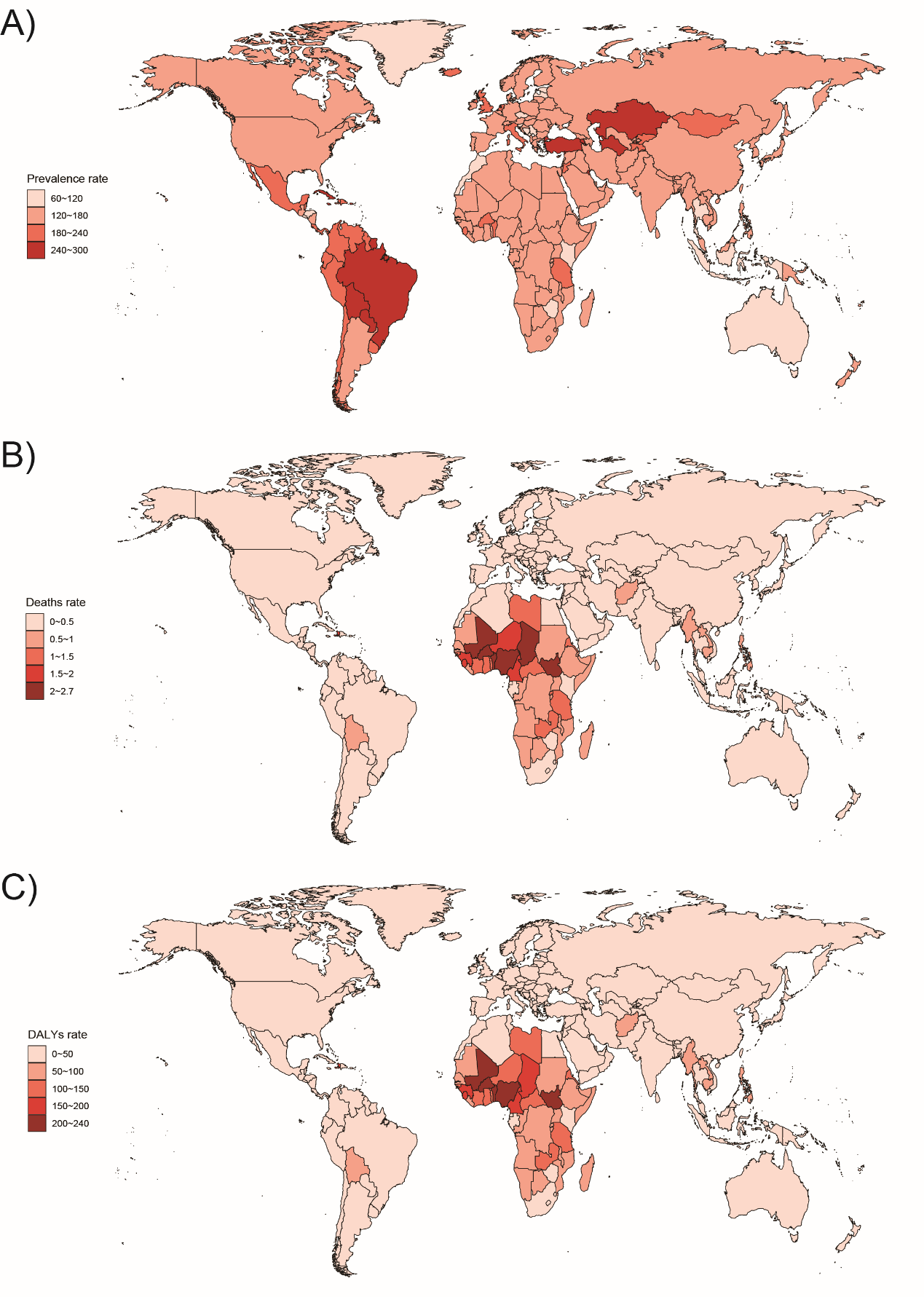
**

**Supplementary Figure 2. Trends in prevalence, mortality, and DALY rates for congenital gastrointestinal malformations in children aged 2-4 years across 204 countries from 1990 to 2021. A) Prevalence rate; B) Mortality rate; C) DALY rate.**

**
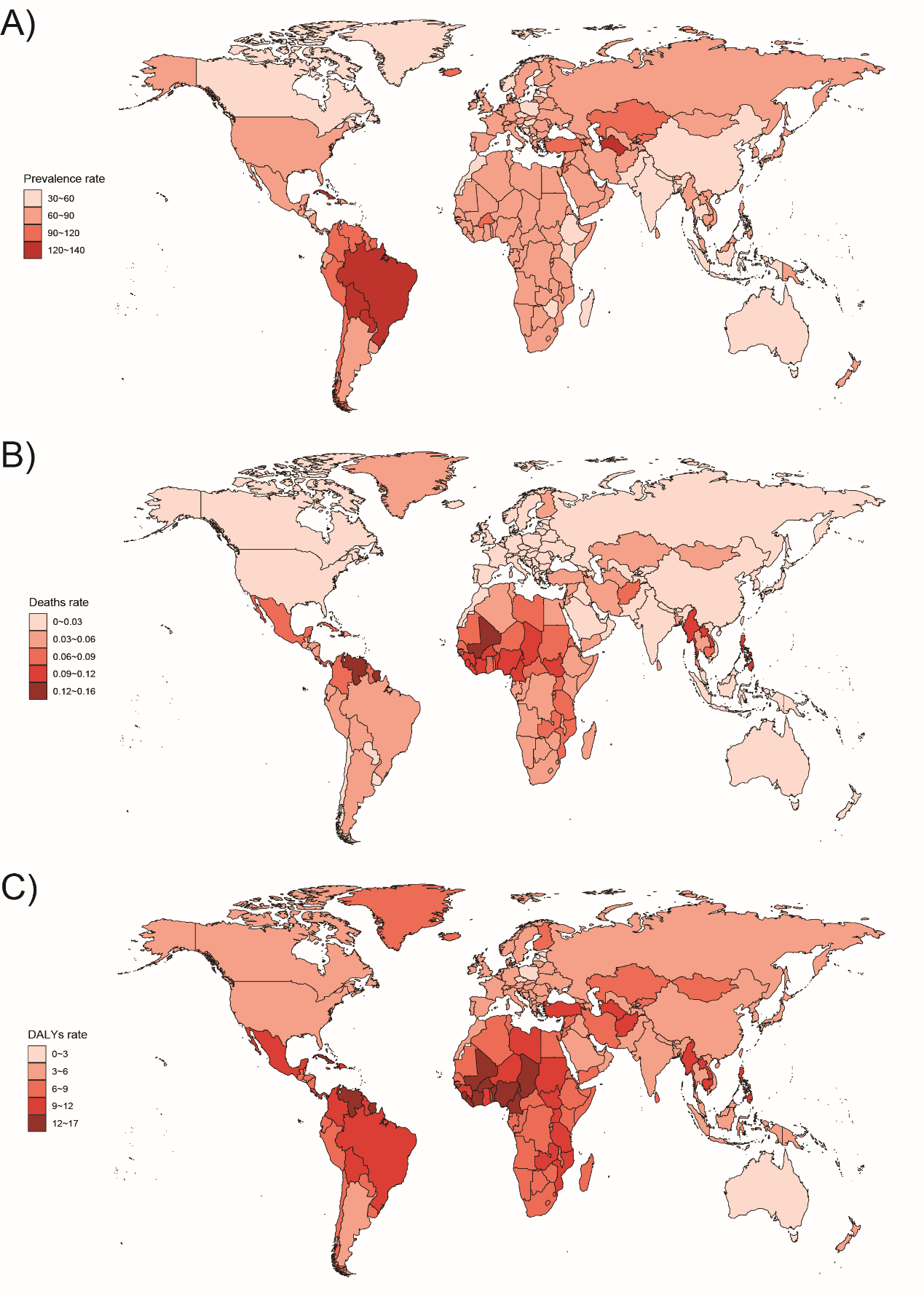
**

**Supplementary Figure 3. Trends in prevalence, mortality, and DALY rates for congenital gastrointestinal malformations in children aged 5-14 years across 204 countries from 1990 to 2021. A) Prevalence rate; B) Mortality rate; C) DALY rate.**

**
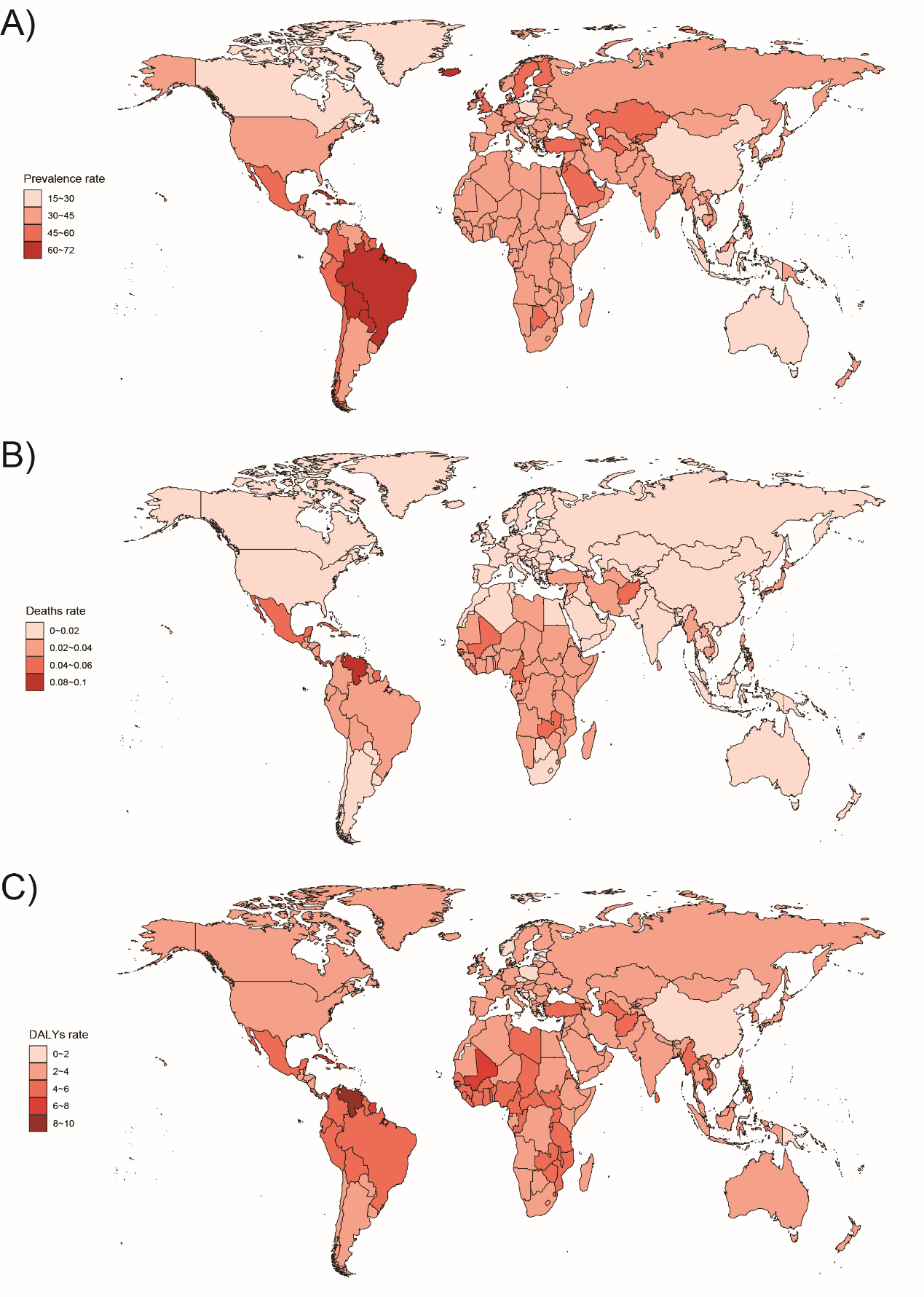
**

**Supplementary Figure 4. Trends in prevalence, mortality, and DALY rates for congenital gastrointestinal malformations in individuals aged 15-19 years across 204 countries from 1990 to 2021. A) Prevalence rate; B) Mortality rate; C) DALY rate.**

**
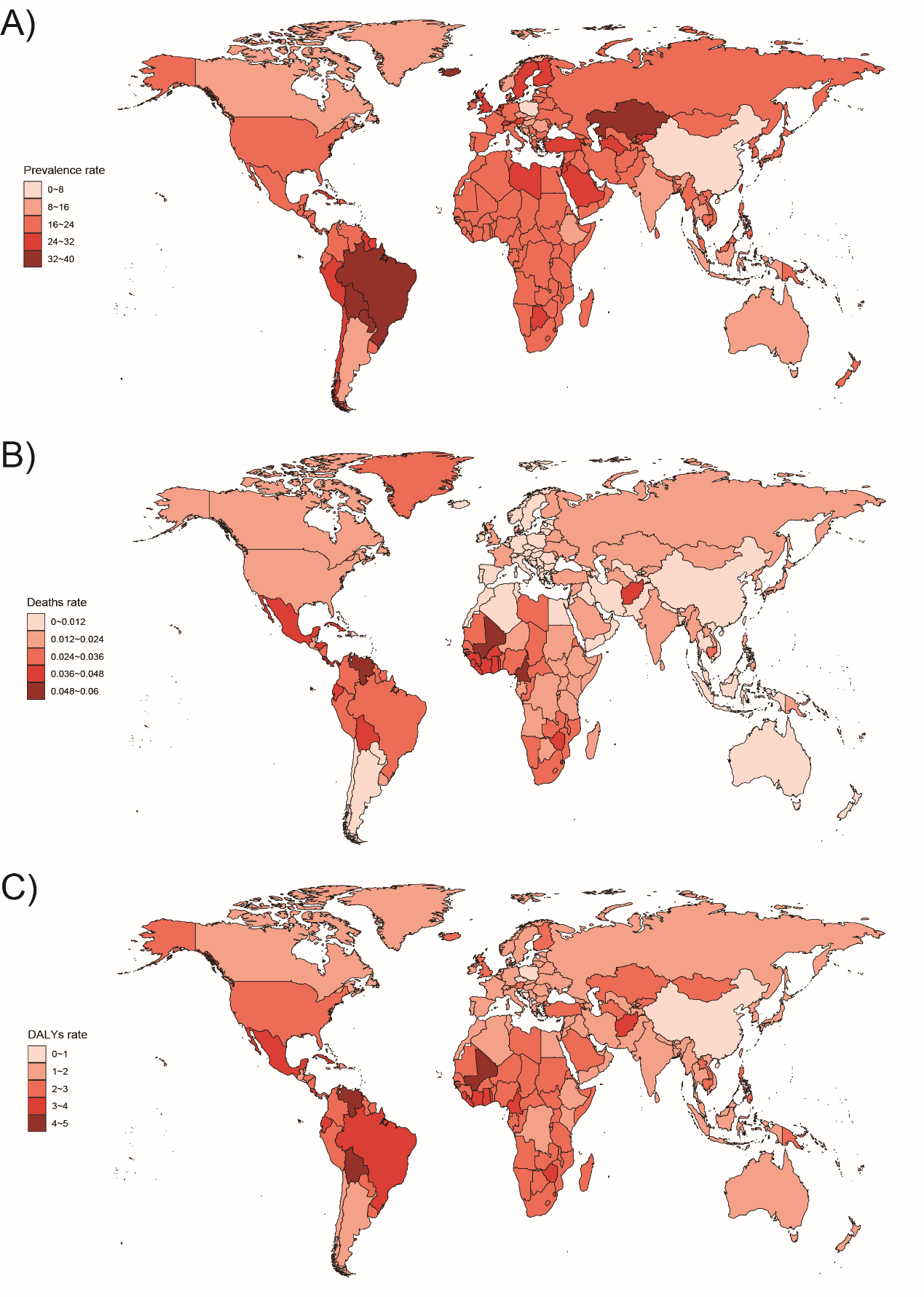
**

**Supplementary Figure 5. Trends in prevalence, mortality, and DALY rates for congenital gastrointestinal malformations in individuals aged 20-54 years across 204 countries from 1990 to 2021. A) Prevalence rate; B) Mortality rate; C) DALY rate.**

**
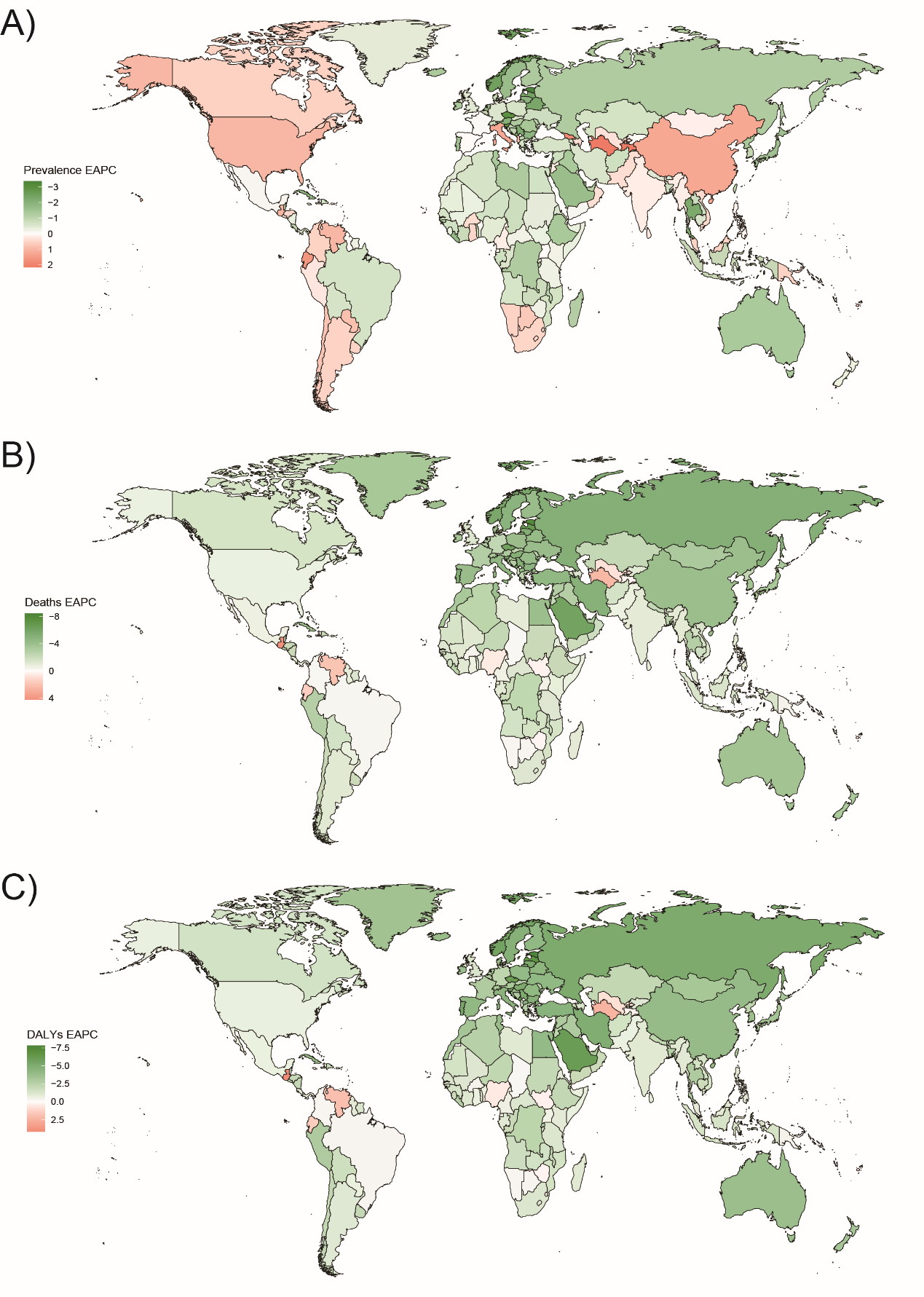
**

**Supplementary Figure 6. National burden of congenital gastrointestinal malformations in children under 1 year of age across 204 countries from 1990 to 2021. A) EAPC of prevalence rate; B) EAPC of mortality rate; C) EAPC of DALY rate.**

**
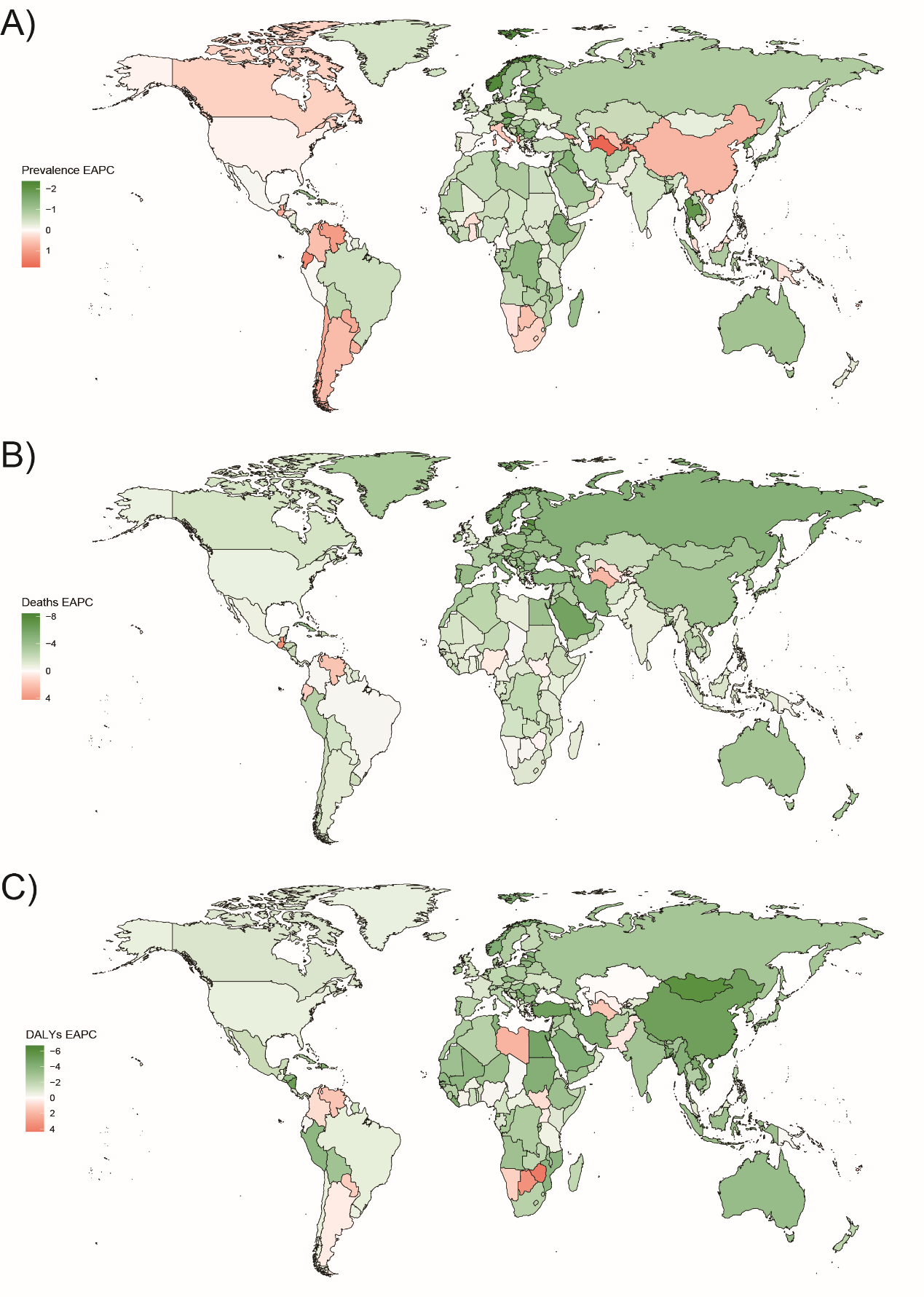
**

**Supplementary Figure 7. National burden of congenital gastrointestinal malformations in children aged 2-4 years across 204 countries from 1990 to 2021. A) EAPC of prevalence rate; B) EAPC of mortality rate; C) EAPC of DALY rate.**

**
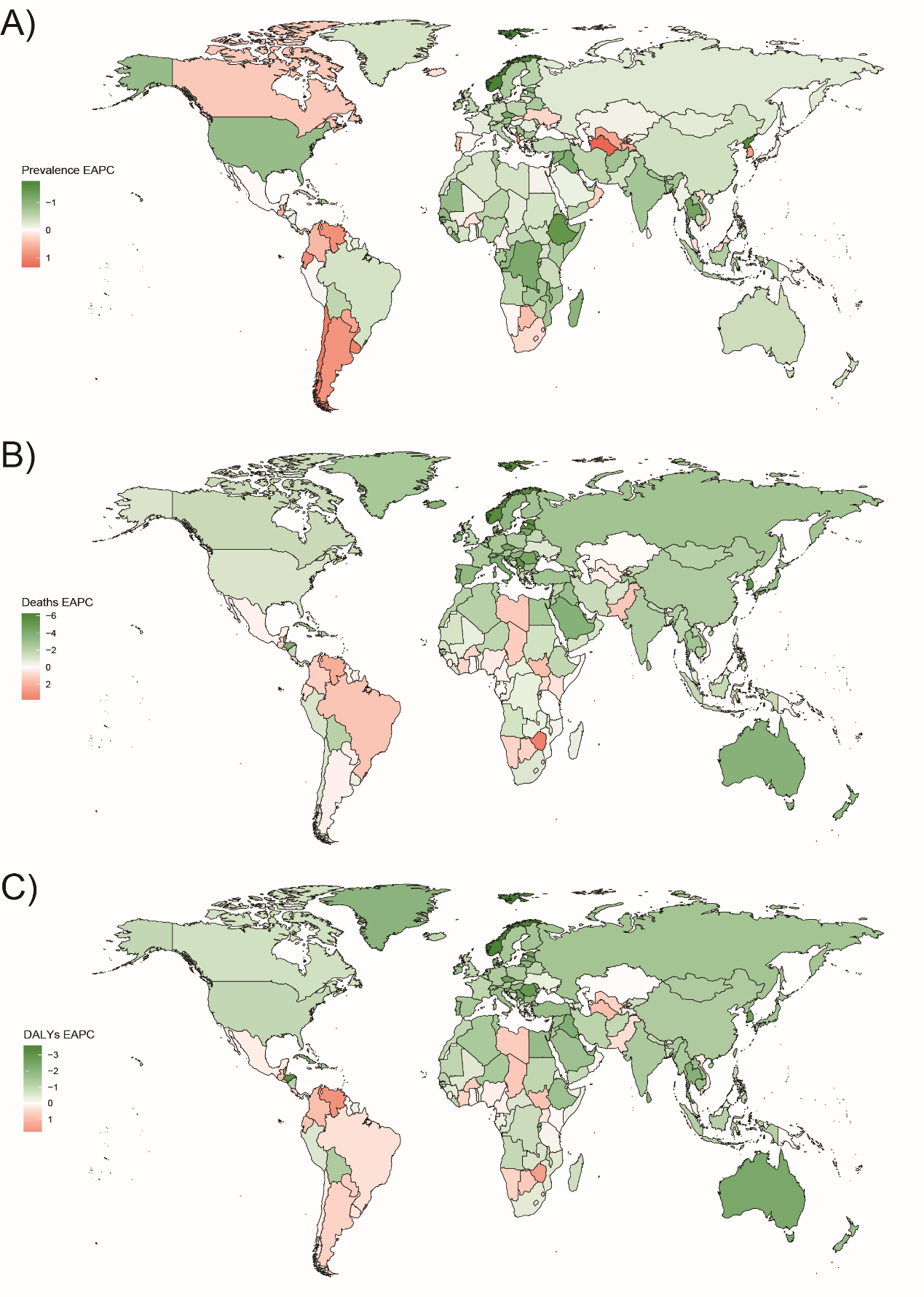
**

**Supplementary Figure 8. National burden of congenital gastrointestinal malformations in children aged 5-14 years across 204 countries from 1990 to 2021. A) EAPC of prevalence rate; B) EAPC of mortality rate; C) EAPC of DALY rate.**

**
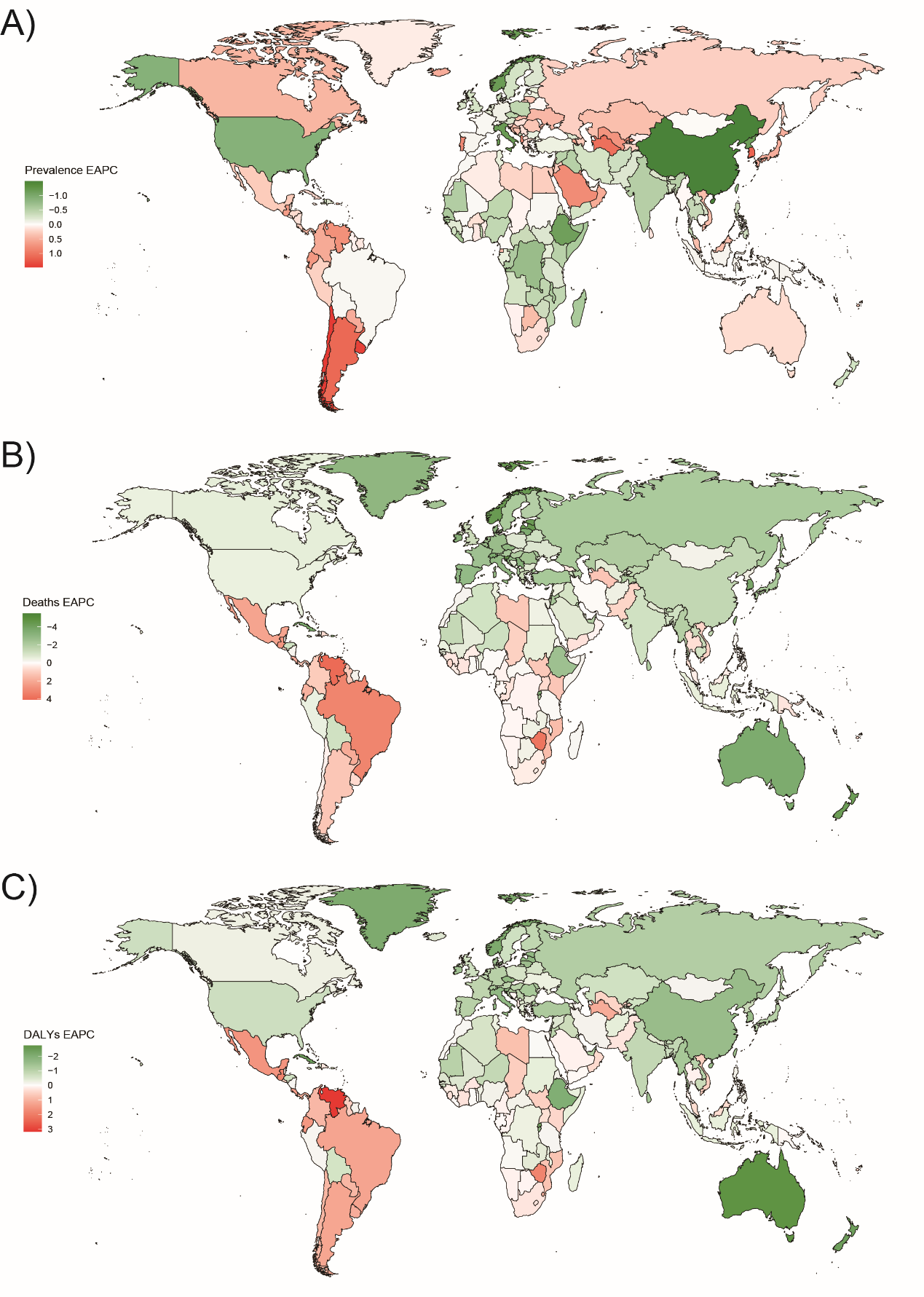
**

**Supplementary Figure 9. National burden of congenital gastrointestinal malformations in individuals aged 15-19 years across 204 countries from 1990 to 2021. A) EAPC of prevalence rate; B) EAPC of mortality rate; C) EAPC of DALY rate.**

**
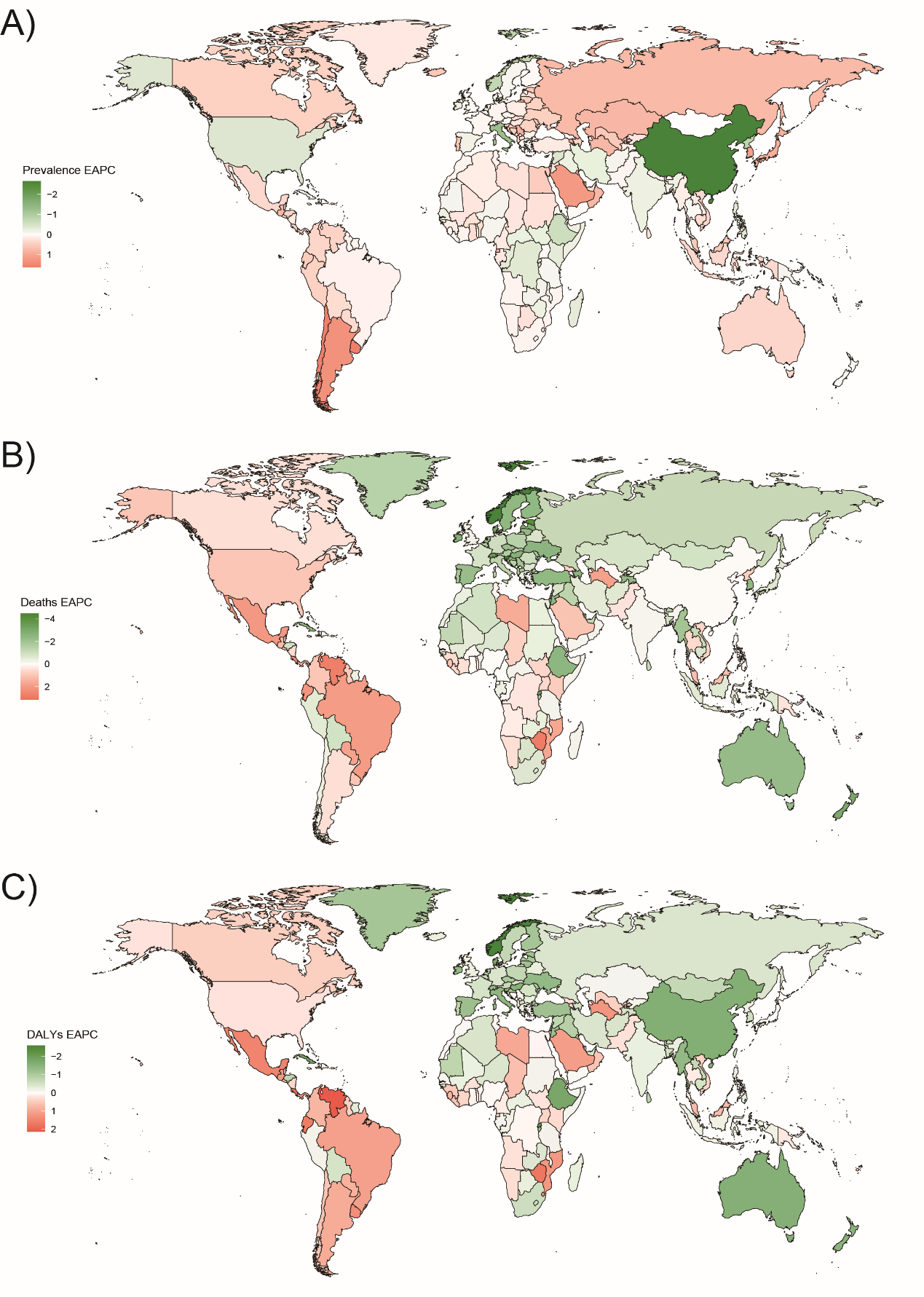
**

**Supplementary Figure 10. National burden of congenital gastrointestinal malformations in individuals aged 20-54 years across 204 countries from 1990 to 2021. A) EAPC of prevalence rate; B) EAPC of mortality rate; C) EAPC of DALY rate.
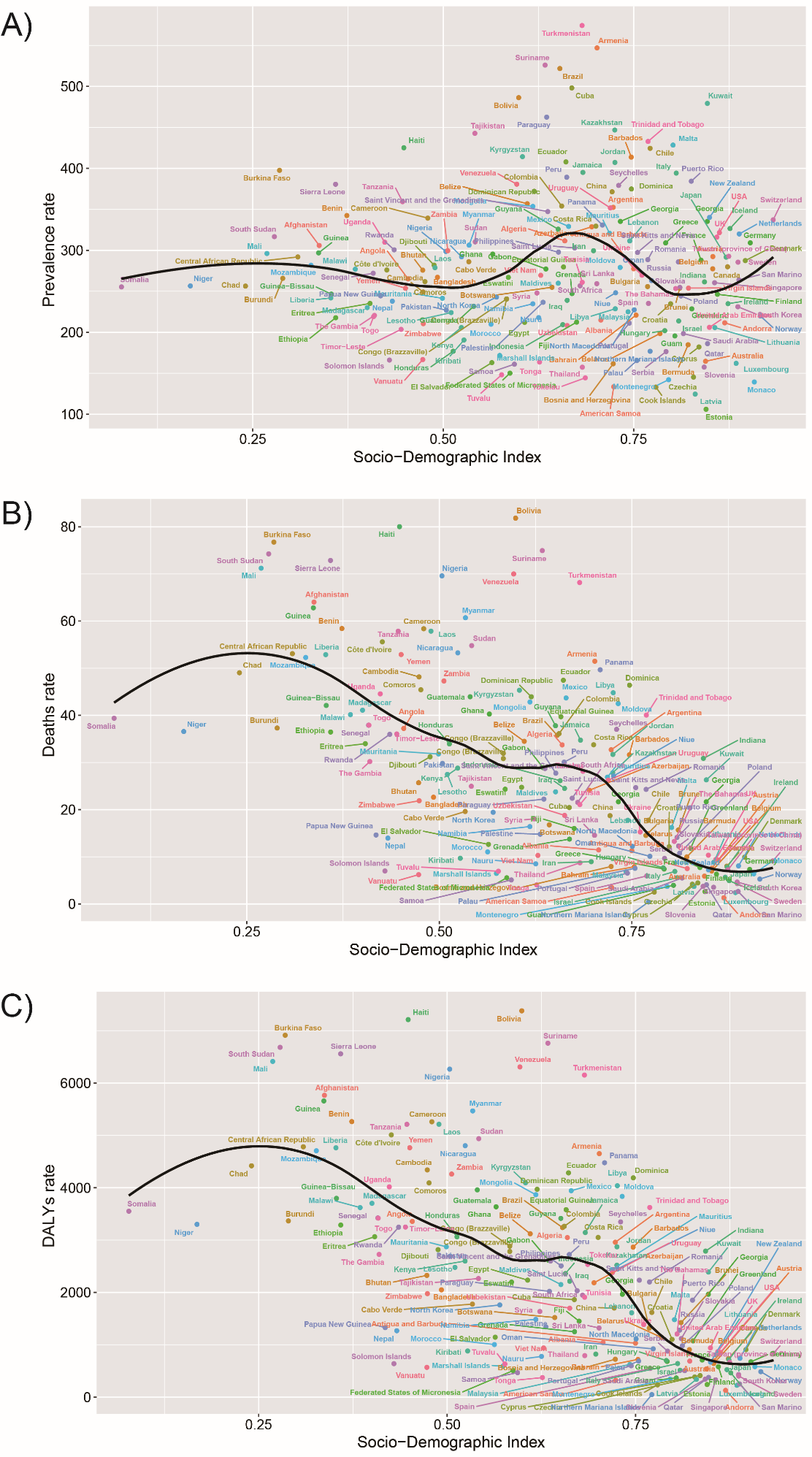
**

**Supplementary Figure 11. Prevalence, mortality, and DALY rates of congenital gastrointestinal malformations in children under 1 year of age across 204 countries in 2021, based on SDI. A) Prevalence rate; B) Mortality rate; C) DALY rate.**

**
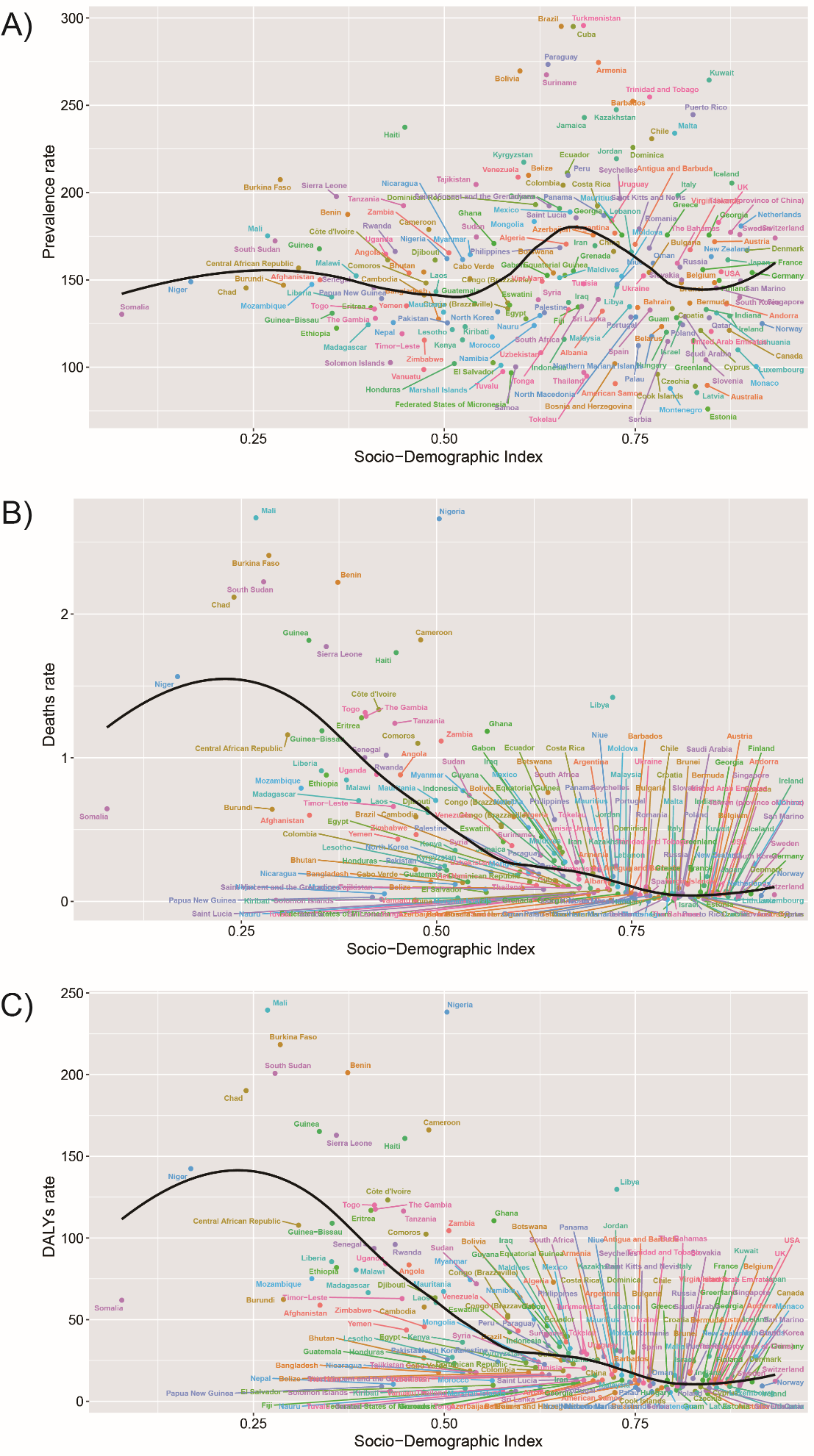
**

**Supplementary Figure 12. Prevalence, mortality, and DALY rates of congenital gastrointestinal malformations in children aged 2-4 years across 204 countries in 2021, based on SDI. A) Prevalence rate; B) Mortality rate; C) DALY rate.
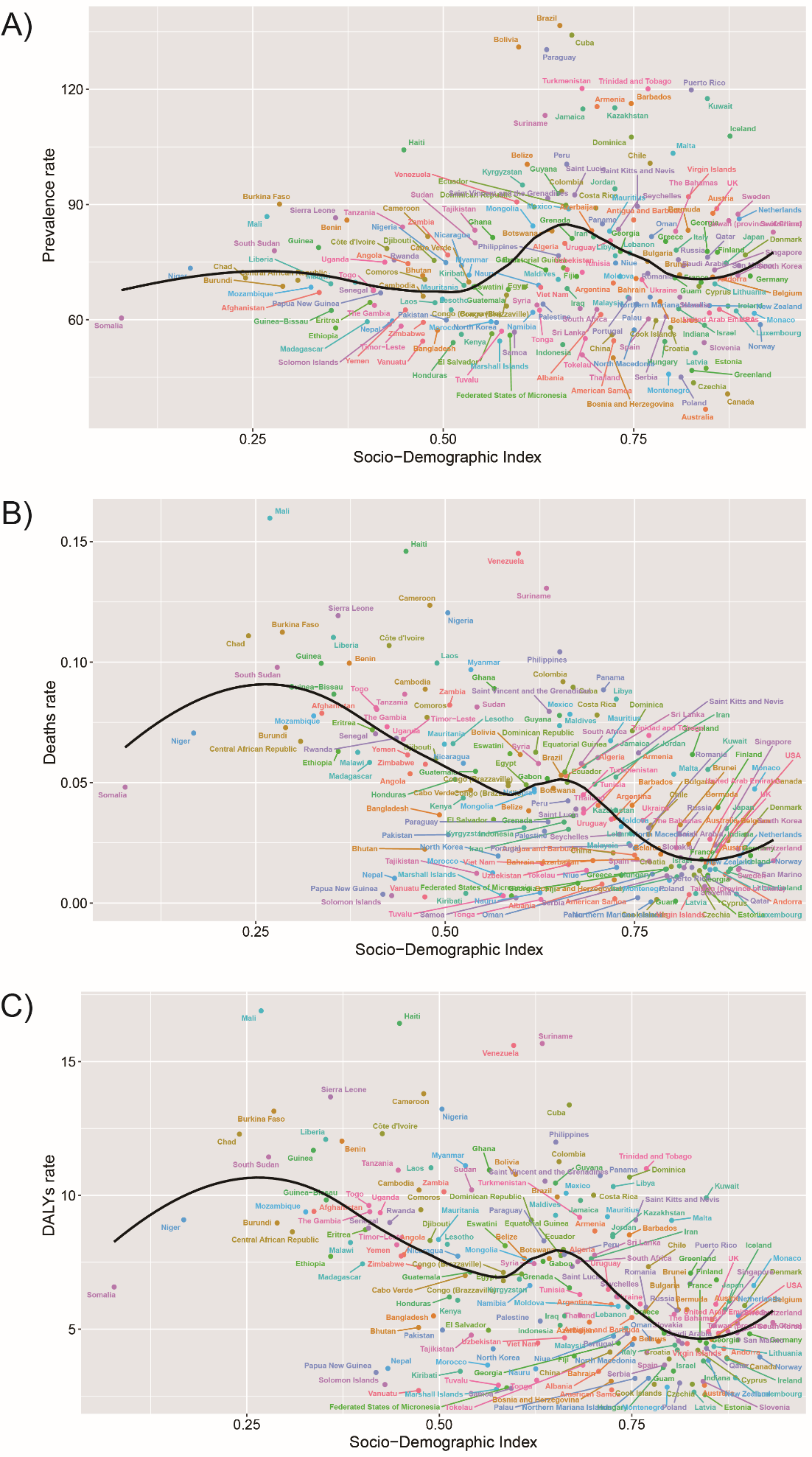
**

**Supplementary Figure 13. Prevalence, mortality, and DALY rates of congenital gastrointestinal malformations in children aged 5-14 years across 204 countries in 2021, based on SDI. A) Prevalence rate; B) Mortality rate; C) DALY rate.
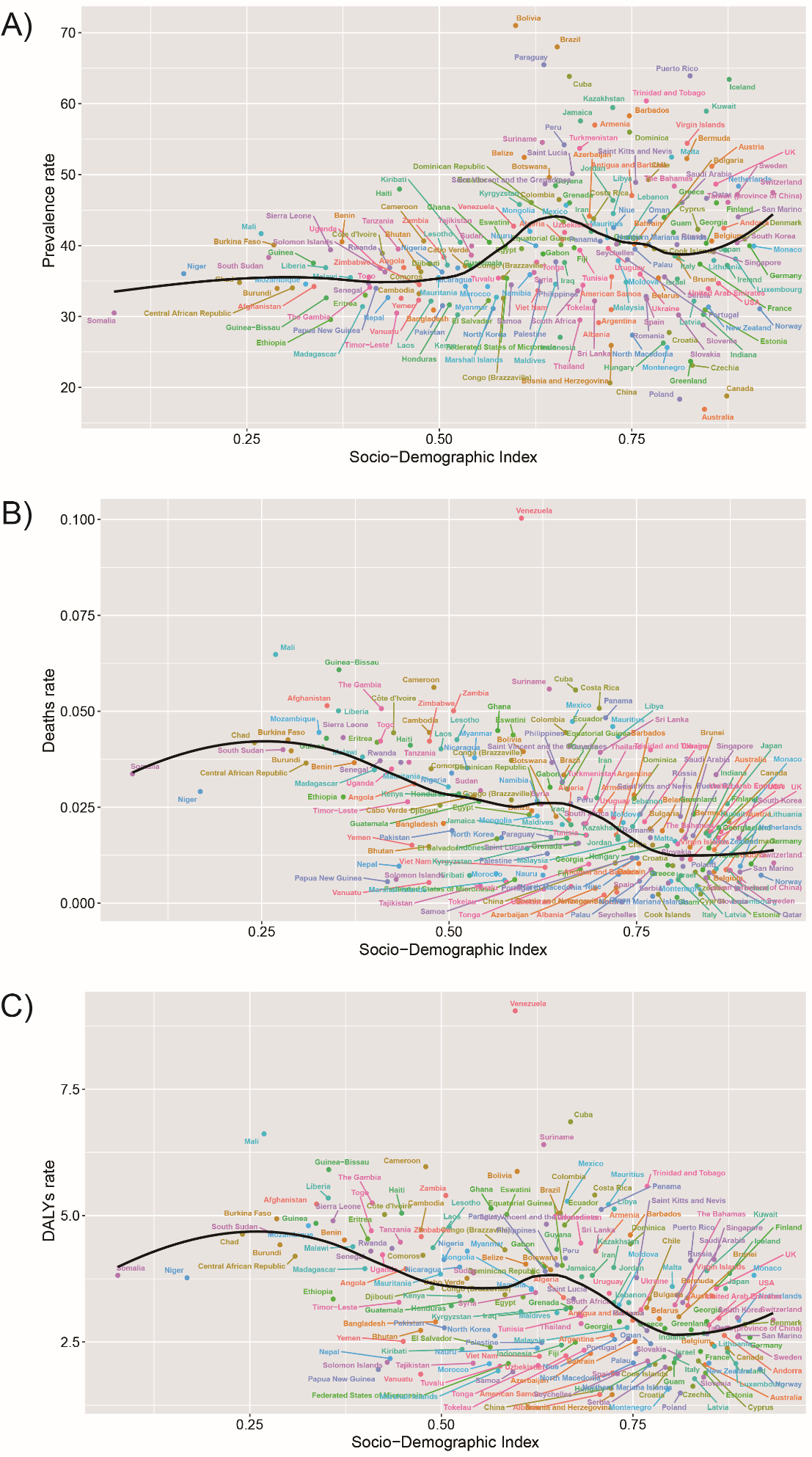
**

**Supplementary Figure 14. Prevalence, mortality, and DALY rates of congenital gastrointestinal malformations in individuals aged 15-19 years across 204 countries in 2021, based on SDI. A) Prevalence rate; B) Mortality rate;C) DALY rate.
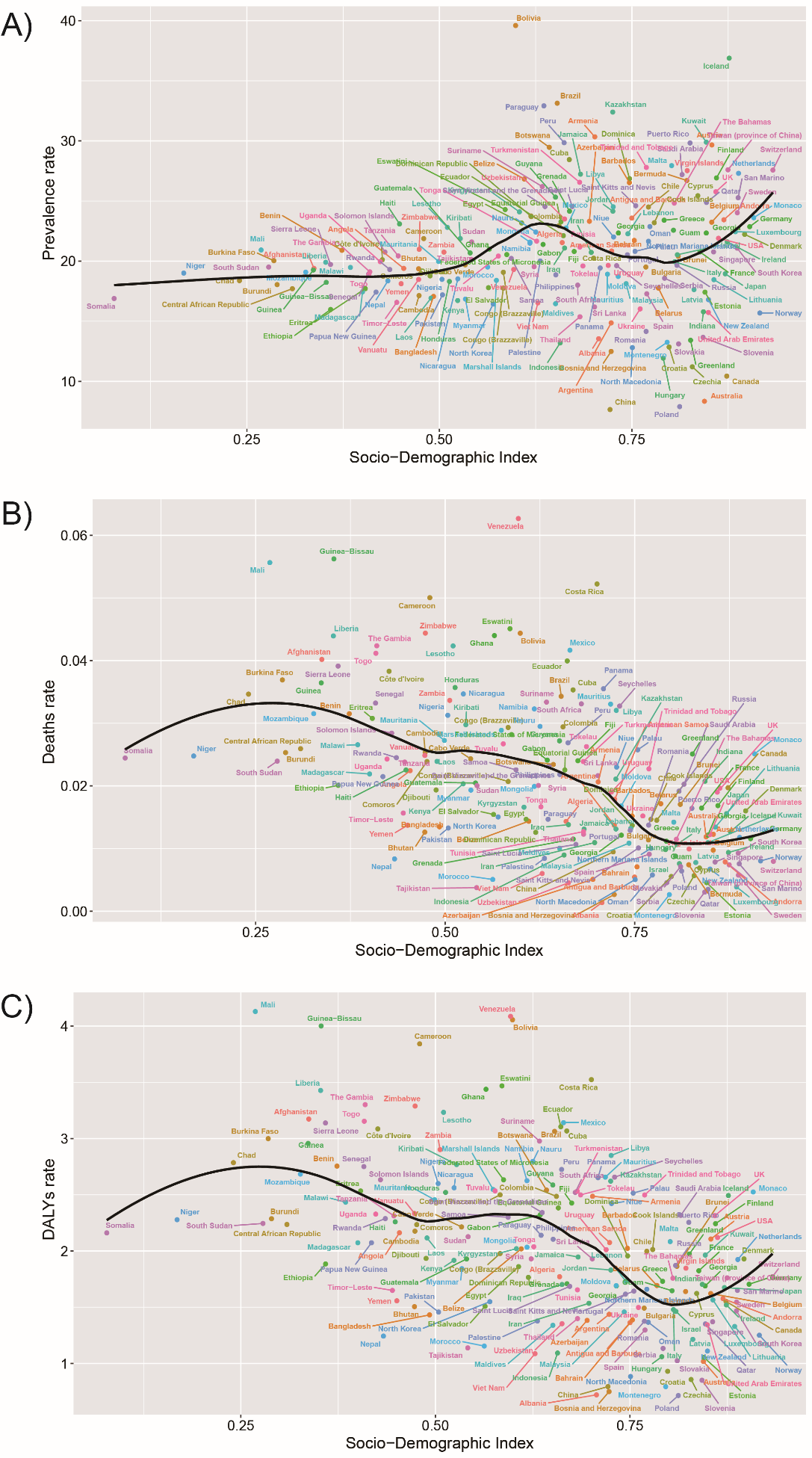
**

**Supplementary Figure 15. Prevalence, mortality, and DALY rates of congenital gastrointestinal malformations in individuals aged 20-54 years across 204 countries in 2021, based on SDI. A) Prevalence rate; B) Mortality rate; C) DALY rate.**
