# Supplementary figures and images for: Global, regional, and national differences in the incidence and mortality of digestive congenital anomalies from 1990 to 2021, with projections for future trends
Source: Front Public Health. 2025 Oct 14;13:1640700. doi: 10.3389/fpubh.2025.1640700 (PMC12558871; doi:10.3389/fpubh.2025.1640700)

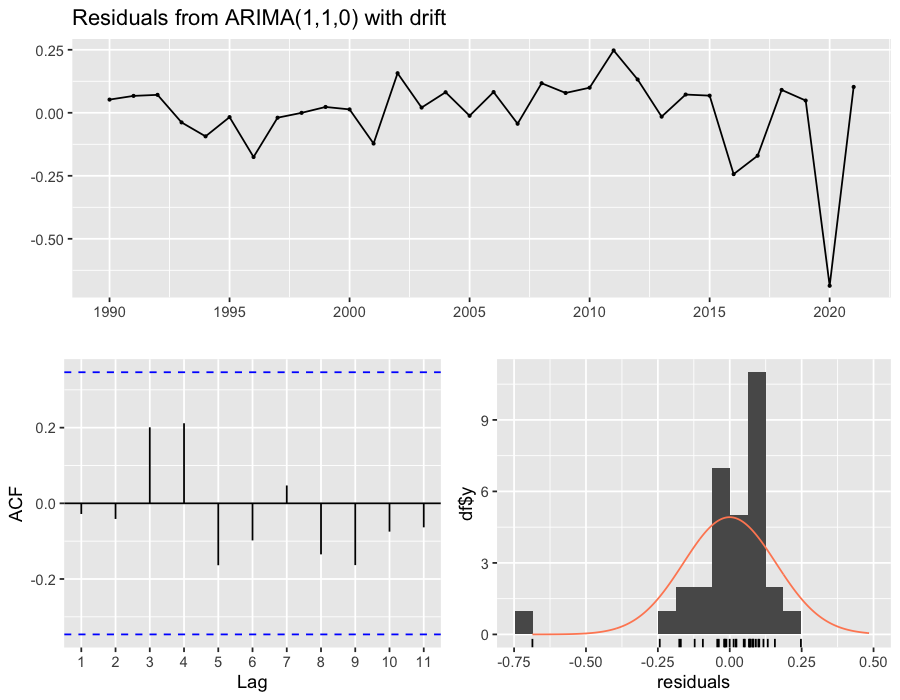

Supplement: Supplementary file 2 [file Data_Sheet_2.zip › Supplementary Information2/model_diagnostics/checkresiduals.png]

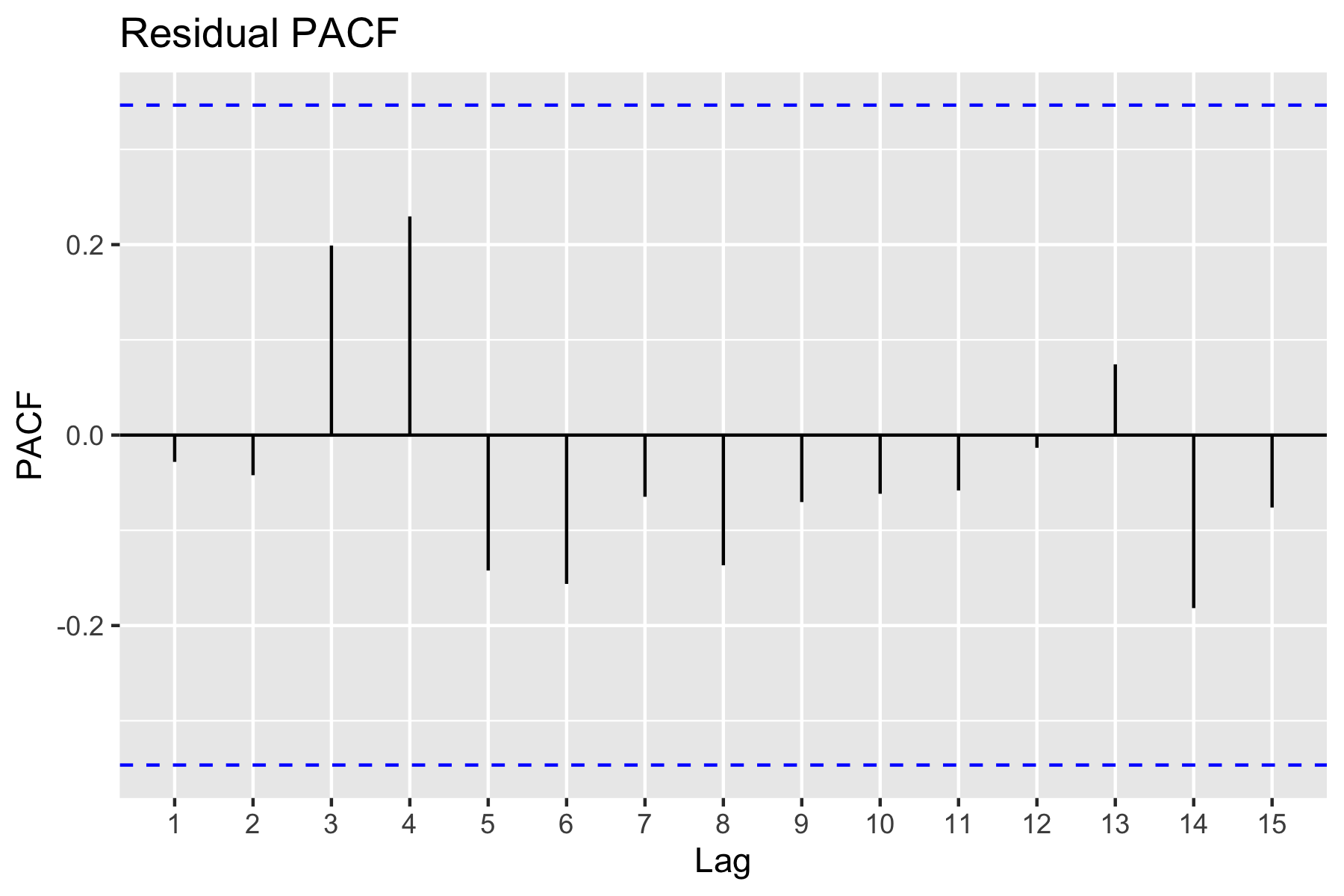

Supplement: Supplementary file 2 [file Data_Sheet_2.zip › Supplementary Information2/model_diagnostics/residual_pacf.png]

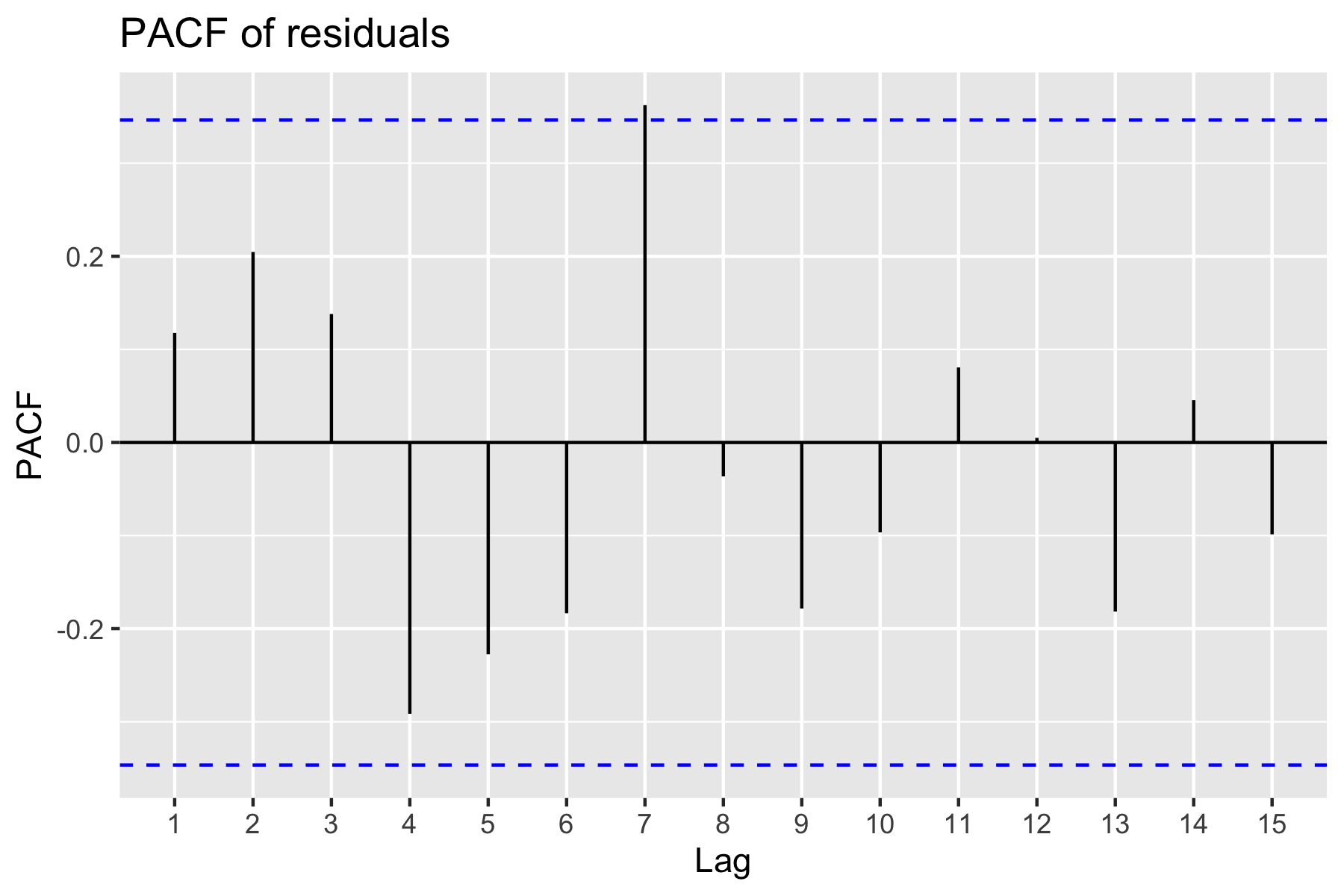

Supplement: Supplementary file 2 [file Data_Sheet_2.zip › Supplementary Information2/model_diagnostics/residuals_pacf.png]

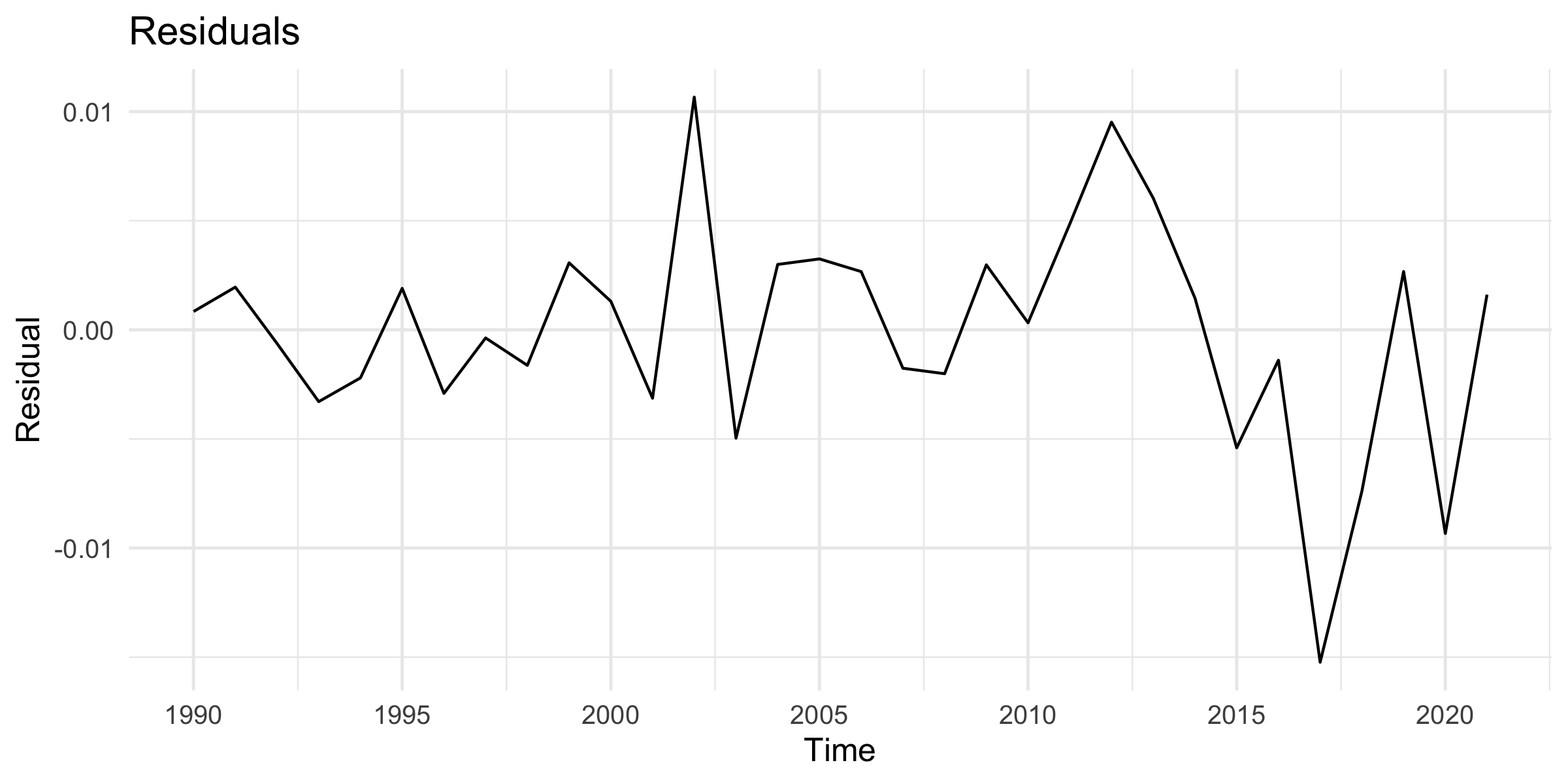

Supplement: Supplementary file 2 [file Data_Sheet_2.zip › Supplementary Information2/model_diagnostics/residuals_series.png]

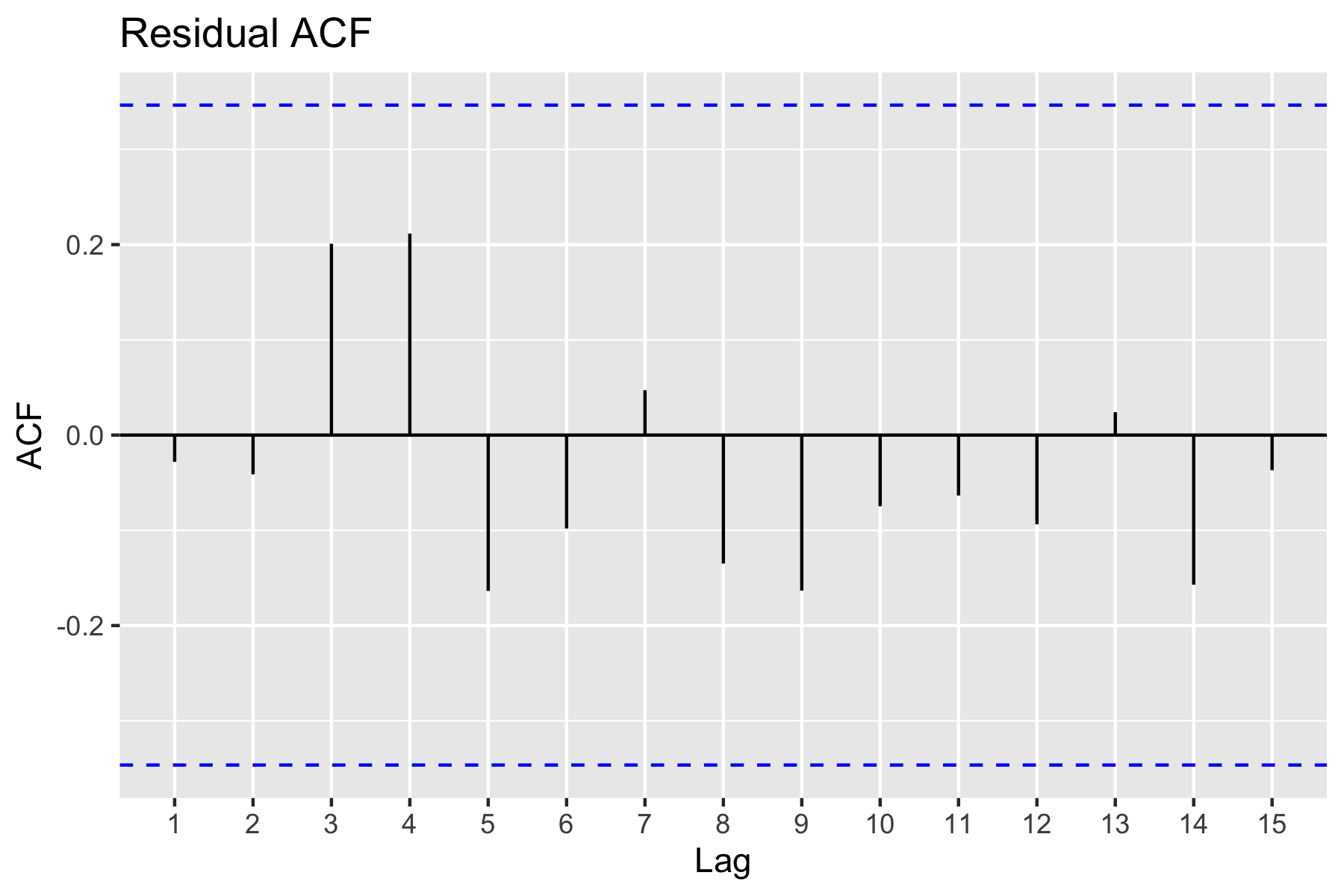

Supplement: Supplementary file 2 [file Data_Sheet_2.zip › Supplementary Information2/model_diagnostics/residual_acf.png]

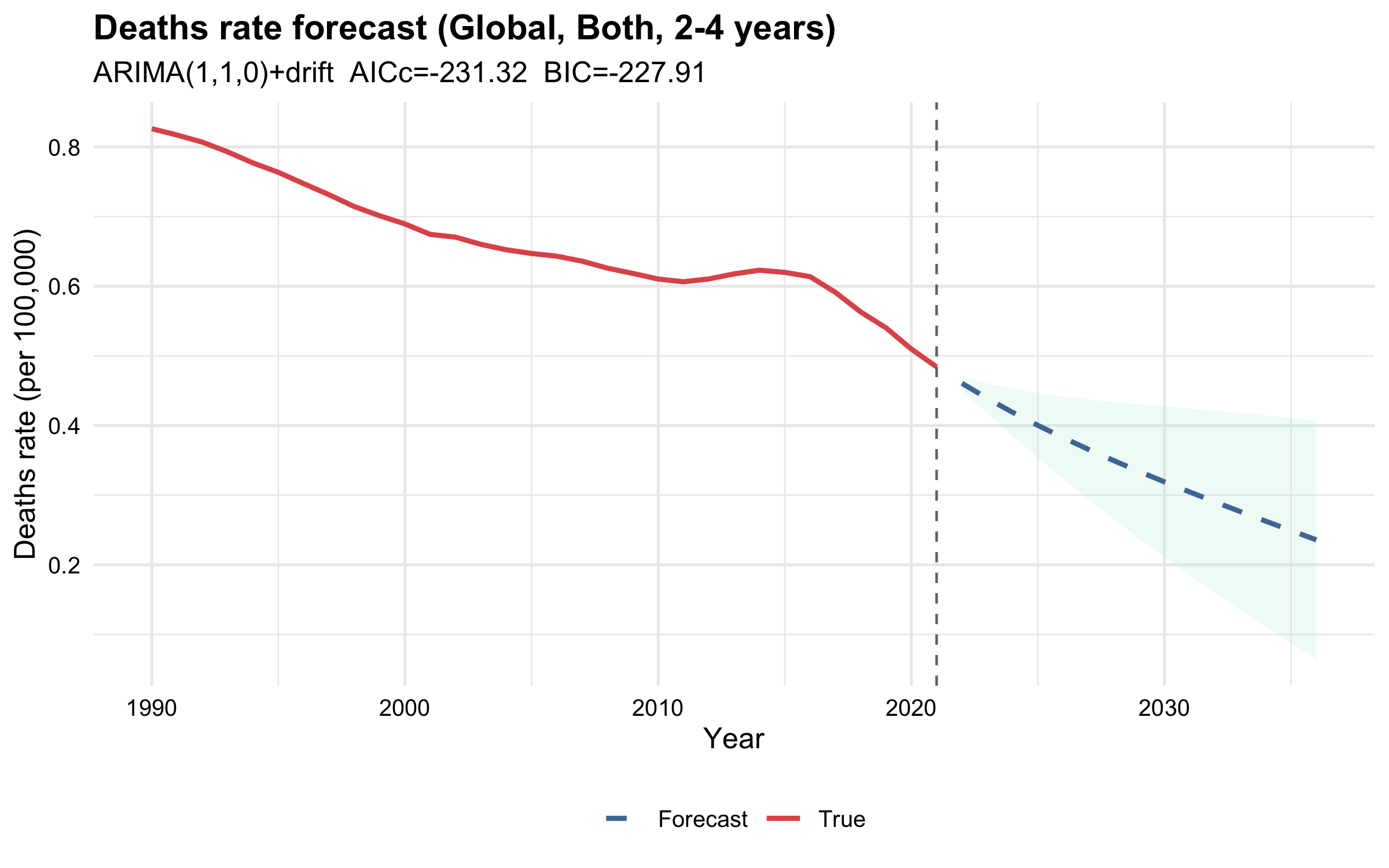

Supplement: Supplementary file 2 [file Data_Sheet_2.zip › Supplementary Information2/model_diagnostics/forecast_plot.png]

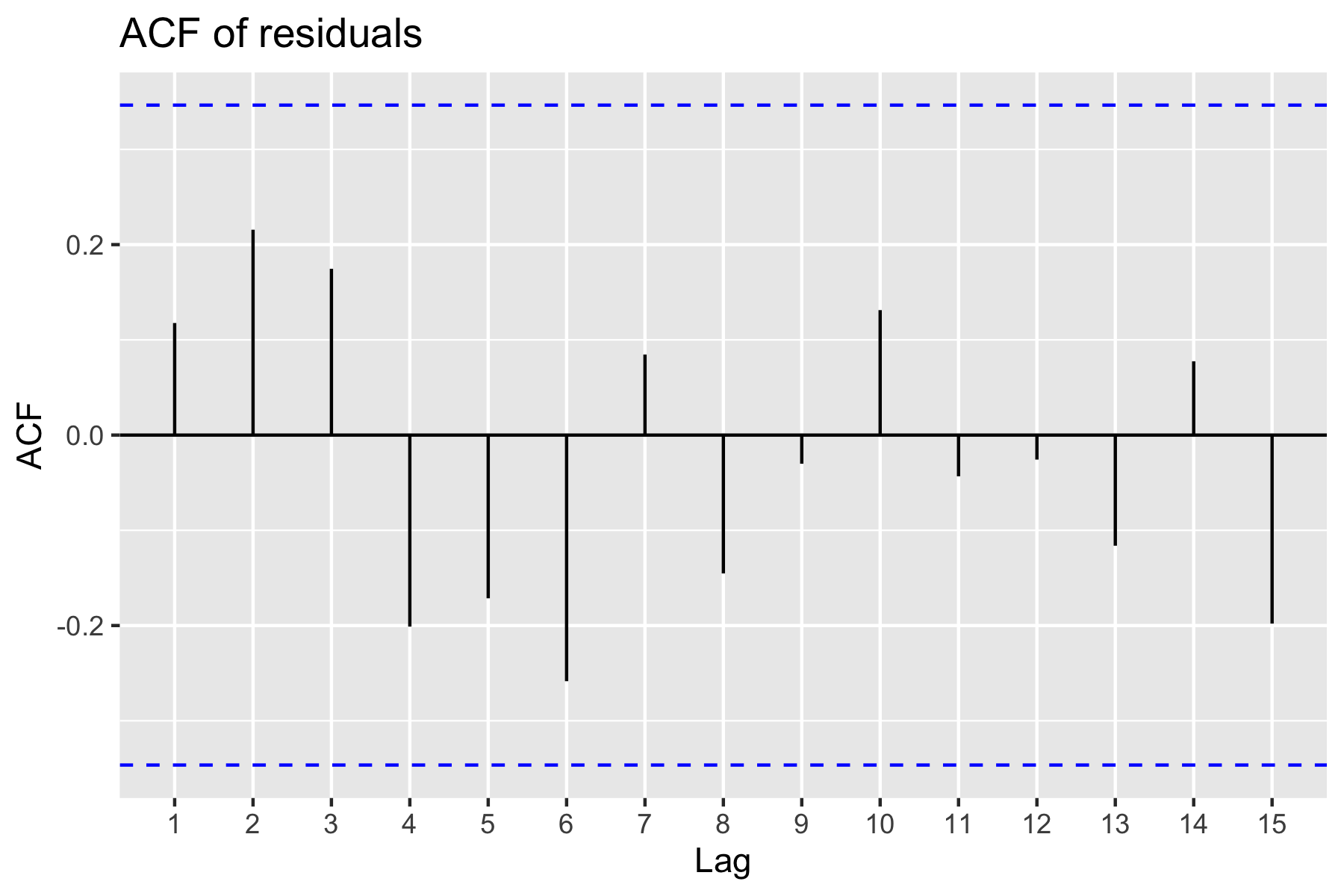

Supplement: Supplementary file 2 [file Data_Sheet_2.zip › Supplementary Information2/model_diagnostics/residuals_acf.png]
